# Establishment of Coral-Bacteria Symbioses Reveal Changes in the Core Bacterial Community With Host Ontogeny

**DOI:** 10.3389/fmicb.2019.01529

**Published:** 2019-07-09

**Authors:** Rachele Bernasconi, Michael Stat, Annette Koenders, Andrea Paparini, Michael Bunce, Megan J. Huggett

**Affiliations:** ^1^Centre for Marine Ecosystems Research, Edith Cowan University, Joondalup, WA, Australia; ^2^Centre for Ecosystem Management, School of Science, Edith Cowan University, Joondalup, WA, Australia; ^3^Faculty of Science, School of Environmental and Life Sciences, The University of Newcastle, Callaghan, NSW, Australia; ^4^School of Veterinary and Life Sciences, Murdoch University, Murdoch, WA, Australia; ^5^Trace and Environmental DNA Laboratory, Department of Environment and Agriculture Curtin University, Bentley, WA, Australia; ^6^Faculty of Science, School of Environmental and Life Sciences, The University of Newcastle, Ourimbah, NSW, Australia

**Keywords:** core bacterial communities, symbiosis, 16S rRNA, metabarcoding, bacterial communities, seawater, sediment, transmission

## Abstract

Bacterial communities are fundamental symbionts of corals. However, the process by which bacterial communities are acquired across the life history of corals, particularly in larval and early juvenile stages, is still poorly characterized. Here, transfer of bacteria of the Scleractinian coral *Acropora digitifera* from adults to spawned egg-sperm bundles was analyzed, as well as acquisition across early developmental stages (larvae and newly settled spat), and 6-month-old juveniles. Larvae were reared under manipulated environmental conditions to determine the source (maternal, seawater, or sediment) of bacteria likely to establish symbiotic relationships with the host using amplicon sequencing of the 16S rRNA gene. Maternal colonies directly transferred bacteria from the families Rhodobacteraceae, Cryomorphaceae, and Endozoicimonaceae to egg-sperm bundles. Furthermore, significant differences in the microbial community structure were identified across generations, yet the structure of the coral bacterial community across early life history stages was not impacted by different environmental rearing conditions. These data indicate that the uptake and structure of bacterial communities is developmentally, rather than environmentally, regulated. Both maternal coral colonies and ubiquitous bacteria found across environmental substrates represent a potential source of symbionts important in establishing the coral microbiome. Uniquely, we report the presence of variation with ontogeny of both the core and resident bacterial communities, supporting the hypothesis that microbial communities are likely to play specific roles within the distinct life history stages of the coral host.

## Introduction

Corals host mutualistic relationships with a dense, dynamic and highly diverse consortium of microorganisms such as dinoflagellate unicellular algae in the family Symbiodiniaceae, bacteria, archaea, viruses and fungi. This complex system has been termed the “coral holobiont" (Rohwer et al., 2002). Although it is well recognized that Symbiodiniaceae are essential for the health of corals and for their success in oligotrophic waters (Fournier, 2013), other components of the holobiont (i.e., bacteria) might equally affect coral fitness. Coral bacterial symbionts contribute to various aspects of coral biology, including nutrition, defence, growth, survival and the general health status (Rosenberg et al., 2007; Bourne et al., 2016; Peixoto et al., 2017). A crucial step in the relationship of corals with their bacterial partners is the establishment, or onset, of the association, whereby bacteria are first acquired by the coral host (Apprill et al., 2009; Sharp et al., 2012; Lema et al., 2014). This critical first association between bacteria and coral early life stages has been attributed to both vertical (maternal) and horizontal (environmental) transmission modes. Vertical transmission of bacterial symbionts occurs in several invertebrates including shallow-water bivalves (Krueger et al., 1996), sponges (Sharp et al., 2007; Lee et al., 2009) and bryozoans (Haygood and Davidson, 1997). This mechanism offers potential for co-diversification and co-evolution of the coral holobiont enabling longevity of these relationships (Munson et al., 1991). Horizontal transmission of symbionts from the environment is also a commonly used mechanism and has been documented in invertebrates such as *Hydra* (Franzenburg et al., 2013), tubeworms (Kikuchi et al., 2007), the broad-headed bug *Riptortus clavatus* (Nussbaumer et al., 2006) and also in the bobtail squid *Euprymna scolopes* where the luminous bacterium *Vibrio fischeri* is acquired (Nyholm and McFall-Ngai, 2004). Both vertical and horizontal transmission modes are likely to be influenced by host reproductive strategies (Apprill et al., 2009, 2012; Sharp et al., 2010, 2012) and several studies suggest that the acquisition of microbial symbionts is consistent with reproductive mode (brooding vs. broadcast spawning) within corals (Hartmann et al., 2017). However, understanding of the mechanisms for acquiring bacterial associations, and how these evolve and are maintained through development stages, is poorly understood.

Recently, deep-sequencing methodologies have been applied to understand the diversity of the bacterial communities associated with specific early life stages of corals (Leite et al., 2017; Zhou et al., 2017). However, these have not yet been applied to document the diversity across development or to track the source (parental or environmental) of bacterial community acquisition. Among broadcast spawning coral only ∼20% acquire their algal symbionts vertically, while the remaining portion take them up from their immediate surrounding environment (Stat et al., 2006; Baird et al., 2009; Quigley et al., 2017). Given their benthic position on the reef, the most likely sources of bacterial partners for corals are the surrounding seawater and/or marine sediment. Seawater and sediment are both characterized by dynamic and diverse free-living bacterial communities, directly available for acquisition via horizontal transfer to the coral host. For corals that acquire Symbiodiniaceae from the environment, this occurs by the larvae, post-settlement and after metamorphosis to a juvenile polyp (Weis et al., 2001). Less information is available on horizontal transmission patterns of bacterial communities (Sharp et al., 2010). Early life stages contain a higher diversity of bacterial taxa in comparison to adult colonies, suggesting that the environment is a reservoir of bacterial symbionts during development (Apprill et al., 2009; Littman et al., 2009a,b; Lema et al., 2014). The main source of bacteria in coral early life history stages is thought to be the water column (Patten et al., 2008; Apprill et al., 2009, 2012). However, sediment bacterial communities are among the most taxonomically diverse within coral reef habitats (Schöttner et al., 2012) and coral mucus has a higher similarity of bacterial taxa with sediment than with the water column (Carlos et al., 2013), thus suggesting that sediments also act as an important source that seed coral microbiomes including symbionts that may play a fundamental role in coral ontogeny and establishment.

Scleractinian corals depend on their associated bacterial symbionts (Rosenberg et al., 2007), however, it is still relatively unclear how the composition and the structure of these associations change through life history stages and which taxa are likely to play a fundamental role. For instance, members of the genera *Burkholderia, Pseudomonas, Acinetobacter, Ralstonia, Inquilinus*, *Bacillus*, *Marinobacter*, *Roseobacter* and the orders *Rhizobiales, Oceanospirellales*, and *Alteromonadales* were ubiquitously found across various coral life stages and were proposed to be important in nitrogen fixation, degradation of the organic sulfur metabolite dimethyl-sulfoniopropionate (DMSP), pollution degradation and production of antibiotics (Nissimov et al., 2009; Raina et al., 2009; Sharp and Ritchie, 2012; Lema et al., 2014; Leite et al., 2017). Some of these taxa were regularly identified as members of the adult coral microbiome (Littman et al., 2009b; Raina et al., 2009; Bourne et al., 2013; Leite et al., 2017), thus suggesting that they may contribute to the formation of the core or resident microbiome (Leite et al., 2017). Another approach that can be applied to elucidate potential bacterial taxa that may play key functional roles is to identify those taxa that occur in very high frequency across individuals of the same host species. This community can be described as the core microbiome and is defined as those taxa that are present in all, or the vast majority, of individuals (Turnbaugh et al., 2007). In coral holobionts “core” and “resident” bacterial communities have been introduced, representing taxa found at high, medium and low frequencies, respectively (Hernandez-Agreda et al., 2018). Therefore, as shown in our study, characterizing bacterial communities based on their frequency and across various developmental stages, may help identify bacterial partners that are important in the development of the coral host.

Given the importance of bacteria in maintaining coral health, it is imperative to understand the establishment of coral-bacterial associations. In this study we documented the associations of bacteria across adult, egg-sperm bundles, larvae, spat and juveniles of the broadcast coral *Acropora digitifera*, one of the dominant species on Ningaloo Reef and widespread across the world (Aeby et al., 2014), using amplicon 16S rRNA gene sequencing. We specifically tracked the vertical transmission of bacteria to spawned bundles of five individual adult colonies. In addition, we experimentally manipulated the exposure of larvae to seawater and sediment to determine horizontal transmission.

## Materials and Methods

**FIGURE 1:**
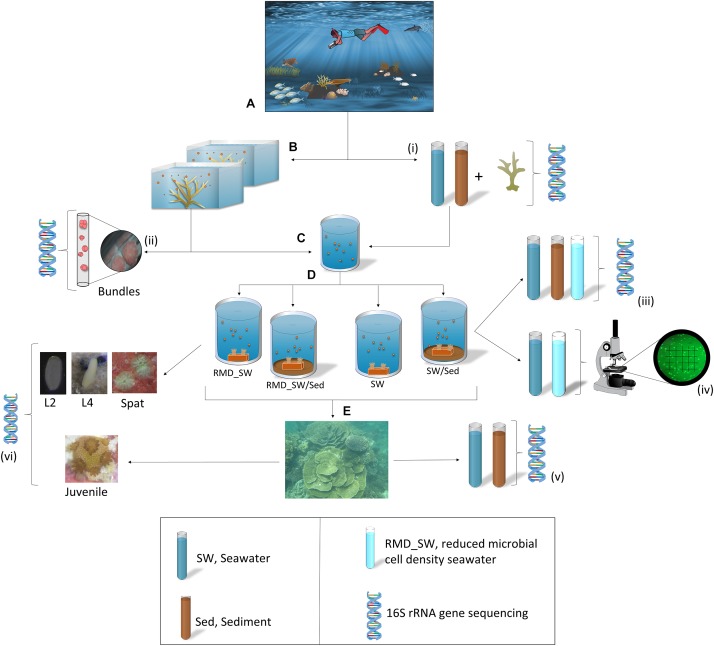
Sampling and experimental design. **(A)** *Acropora digitifera* adult coral colonies were relocated from the reef to the Coral Bay jetty. **(B)** A few hours prior to spawning colonies were placed into individual tubs containing seawater. **(C)** Egg–sperm bundles were collected for fertilization, and **(D)** 8 h old larvae were transferred to four experimental treatments for larval rearing: RMD_SW, reduced microbial cell density seawater; RMD_SW/Sed, reduced microbial cell density seawater and sediment; SW, seawater, SW/Sed, seawater and sediment. Seawater (SW) and sediment (Sed) samples were collected from the collection site and used in tanks during the manipulative experiment. **(E)** Once settled onto terracotta tiles, newly settled spat were deployed back onto the reef for 6 months. **(i–iii,v,vi)**: DNA symbols indicates where various samples (*n* = 5 in all cases) were taken for 16S rRNA gene sequencing. **(iv)** Samples of SW and RMD_SW (*n* = 3) were preserved in 2% formalin at 4°C and bacterial density was determined via microscopy. Symbols are from the Integration and Application Network (ian.umces.edu/symbols/).

Colonies of the Scleractinian coral *A. digitifera* were collected from Ningaloo Reef in Coral Bay, Western Australia (23°10.698′S, 113° 45.634′E) 3–5 days prior to predicted spawning. Eight healthy colonies (Supplementary Figure 1) were collected from the reef and transported to the Coral Bay jetty (Figure 1A). A few hours prior to spawning, colonies were placed into individual 60 L tubs containing seawater (Figure 1B and Supplementary Figure 1I). All colonies spawned at 2200–2300 h on 21 March 2017. Egg–sperm bundles were gently mixed in 20 L of seawater for fertilization (Figure 1C) and after 3 h, embryos were rinsed with 0.22 μm filtered seawater (FSW).

In order to characterize their bacterial communities, adults and bundles were collected and snap frozen in liquid nitrogen. For adults, fragments of each colony (three replicate tissue subsamples, ∼1 cm^2^ each) were collected prior to spawning (Figure 1i). Bundles were collected during spawning, from five coral colonies, placed in a cell strainer (40 μm nylon mash, Scientific Laboratory supplies), and rinsed in FSW prior to freezing (Figure 7ii).

### Microbial Acquisition Experiment

Approximately 8 h old larvae were randomly assigned to one of four treatments (Figure 1D), each with five replicates (20 acid washed tanks in total). The treatments were designed to manipulate larval exposure to microbial communities from sediment and seawater and were: (1) reduced microbial cell density seawater (RMD_SW, filtered at 0.22 μm), (2) reduced microbial cell density seawater + sediment (RMD_SW/Sed), (3) seawater (SW) and, (4) seawater + sediment (SW/Sed). To establish the treatments, seawater was collected from the ocean surface while sediment was collected with 50 mL sterile tubes by scooping the oxic layer (∼1 cm depth) within 1 m from the coral colonies. Five replicate sediment (1.8 mL each), and seawater (2 L each, filtered on sterivex filters) samples were snap frozen in liquid nitrogen for bacterial analysis (Figure 1i).

For the experiment, the tanks each contained 2 L of seawater (either reduced or entire microbial density) and those with sediment had 50 mL of sediment thinly covering the bottom. Sediment was added to the tanks 7 h before the experiment started in order to allow it to completely settle. Sediment, seawater, and reduced microbial cell density seawater samples were snap frozen in liquid nitrogen for bacterial analysis (*n* = 5, Figure 1iii). The temperature was maintained at 25°C to match the daily mean local water temperature (25.1°C). After 24 h a gentle airflow was added to each tank. Larvae were maintained at ∼1 larva mL^-1^ until fully competent (Tebben et al., 2011), approximately 5 days after fertilization. One pre-conditioned terracotta tile was placed inside each tank to enable larval settlement (Figure 1D). Each tile was labeled, and then tiles were removed from tanks and randomly stacked with a ∼0.5 cm gap between tiles and deployed for 6 months onto the reef where adult coral colonies were collected (Figure 1E). Five replicate sediment and seawater samples from the deployment site were collected and snap frozen in liquid nitrogen for bacterial analysis (Figure 1v). Twenty larvae (days 2 and 4) and 20 spat (day 7) were collected from each tank and 10 juveniles (6 months) were collected from each tile (Figure 1vi). Samples were rinsed in FSW, and snap frozen in liquid nitrogen for 16S rRNA gene sequencing analysis. Samples of seawater in the SW and RMD_SW treatments (*n* = 3) were preserved in 2% formalin at 4°C and bacterial density was determined via microscopy. Cells were filtered onto 0.22 μm membranes, stained with SYBR Gold and enumerated under epifluorescence microscopy. Ten fields of view were counted for each sample (Figure 1iv).

### Ethics Statement

Permission to conduct the fieldwork and collect coral samples was granted by the Western Australian Department of Parks and Wildlife (permit#SF010989) and Western Australian Department of Fisheries (permit#2895). No special animal ethics approval was needed.

### Microbial Community Characterization: MiSeq Illumina Analysis of 16S rRNA Gene

Prokaryotic diversity for all samples (Figure 1) was assessed through 16S rRNA gene sequencing using an Illumina MiSeq platform (Illumina, San Diego, CA, United States) at Curtin University, Perth (Australia). Mixed genomic DNA was extracted from coral samples using the PowerPlant DNA Isolation Kit (MoBio, Solana Beach, CA, United States), following Sunagawa’s modifications for maximum yield (Sunagawa et al., 2010). Mucus and tissue were removed from the adult coral fragments with an air gun and 1X PBSE pH 7.4, while early life stages were added directly into the extraction tubes. Mixed genomic DNA was extracted from water and sediment samples with the PowerSoil DNA Isolation Kit (MoBio, Solana Beach, CA, United States), following the manufacturer’s instructions. Sediment samples where purified using AMPure XB Beads (Agencourt, Beckman Coulter, United States) following the manufacturer’s instructions.

Bacterial communities were sequenced using barcoded primers 515F (F: 5′-GTGCCAGCMGCCGCGGTAA G-3′) and 806R (R: 5′-GGACTACHVGGGTWTCTAAT-3′) for the V4 variable region of the 16S rRNA gene (Caporaso et al., 2012). Quantitative polymerase chain reaction (qPCR) was used to determine the optimal yield of DNA to be added to each PCR reaction (Murray et al., 2015). Amplicons were produced using a single round of PCR with fusion tag primers consisting of Illumina adaptor regions, MID tags unique to each sample and the 16S rRNA template specific primers. Reagents included 1× AmpliTaq Gold Buffer (Life Technologies, Carlsbad, CA, United States), 2 mM MgCl2, 0.25 μM deoxyribonucleotide triphosphate (dNTPs), 10 μg Bovine Serum Albumin (BSA), 5 pmol of each primer, 0.12 × SYBR Green (Life Technologies), 1 Unit AmpliTaq Gold DNA polymerase (Life Technologies), 2 μL of DNA and UltrapureTMDistilled Water (Life Technologies) to 25 μL. PCRs were run on Applied Biosystems StepOnePlus Real-Time PCR: initial denaturation at 95°C for 5 min, 35 cycles of 30 s at 95°C, 30 s at 50°C, and 45 s at 72°C, with a final extension for 10 min at 72°C. Prior to library pooling, duplicate PCR products from each sample were combined. Extraction controls containing no samples, and PCR negative controls, were added to sequencing runs to assess contamination, and taxa detected within them were excluded from analyses (Supplementary Data 1).

Libraries were prepared by pooling amplicons from each sample in equimolar ratios determined by quantification on a Labchip R GX Touch HT (Perkin Elmer, Waltham, MA, United States). Final libraries were size selected using a Pippin Prep (Sage Science, Beverly, MA, United States), purified using the Qiaquick PCR purification Kit (Qiagen, Venlo, Netherlands) and sequenced unidirectionally using Illumina 300 cycle MiSeq V2 Reagent Kits and standard flow cells on an Illumina MiSeq platform.

### Bioinformatics Analysis of MiSeq Illumina Sequencing Outputs

Raw sequence data was extracted with Geneious version 8.1.7^1^ (Kearse et al., 2012). Using 100% matches the adaptor, MID tags and primer sequences were removed. De-noising and quality filtering was performed using Quantitative Insights Into Microbial Ecology, Mac version 1.9.1 (MacQIIME) (Caporaso, 2011). Short sequences (<200 bp), sequences containing ambiguous base calls or homopolymer runs above 6 bp were discarded. Chimeras were removed using ChimeraSlayer (Haas et al., 2011). The remaining sequences were clustered into Operational Taxonomic Units (OTUs) based on an open-reference OTU picking method (Rideout et al., 2014) at 99% identity using UCLAST (Edgar, 2010). This method was used in order to keep sequences that are not perfectly matched to the GreenGenes reference database release 13_8 (DeSantis et al., 2006), therefore preventing the loss of novel OTUs. Alignment was done with PyNAST version 1.2.2 (Caporaso et al., 2010), and taxonomy was assigned to OTUs using the RDP classifier (Wang et al., 2007), against the GreenGenes database. Non-bacterial sequences (i.e., Archaea, Eukarya, chloroplast, and mitochondria) and absolute singletons (OTUs that occur only once in the dataset) were removed in a post-OTU picking step.

To avoid the analysis of rare sequences possibly generated by sequencing error, OTUs present in less than 0.01% of the total abundance in the table were excluded. This filter reduced the quantity of phylotypes from 18,051 to 973 OTUs. The resulting dataset contained 663,578 high quality sequences, ranging from 584 to 14,407 sequences per sample. To avoid biases generated by unequal sampling depth, sequences were rarefied to an even number (*n* = 831) which corresponded to the sequence depth present in the sample with the third lowest number of sequences, resulting in 962 OTUs (Supplementary Data 2) which were used in all downstream analyses.

MacQIIME and excel were used to explore the dynamics of core and resident bacteria associated with *A. digitifera* development stages. Bacterial phylotypes consistently reported in >80% of the samples within each development stage were considered the “core bacterial communities,” bacteria phylotypes present in 50–79% of the samples within each life stage were considered “resident,” following Ainsworth et al. (2015) and Hernandez-Agreda et al. (2016).

Alpha diversity calculations of the microbial communities, along with relative abundance summaries, were performed with MacQIIME using alpha_diversity.py, and summarize_taxa_through_plots.py as well as excel, while Venn Diagrams were built using MetaComet (Wang et al., 2016). Alpha diversity metrics included number of OTUs, Chao1 (Chao, 1984), and Shannon indices (Shannon, 1948) were each compared between early life stages (L2, L4, spat, and juvenile) using the non-parametric Kruskal–Wallis test in SPSS Version 25.0.

### Data Availability

Sequence data were deposited and are publicly available in the NCBI Sequence Read Archive (SRA) under the BioProject ID PRJNA526947, accession numbers SAMN11097120 – SAMN11097248 and SAMN11603544-SAMN11603546.

### Statistical Analysis

Sorenson (Bray-Curtis) distance was used on the final fourth root transformed dataset to construct a resemblance matrix and non-metric multidimensional scaling (nMDS) was used to visualize patterns in microbial community structure among treatments and development stages. PERMANOVA was used to examine the differences in microbial assemblages between treatments, and across development stages. Prior to PERMANOVA analyses data were tested to ensure equal dispersion using a resemblance-based permutation test (PERMDISP). As the PERMDISP assumptions were not met (*p* < 0.05), the PERMANOVA significance level was set to 0.01 as a precaution. A similarity percentage (SIMPER) was used to determine the key contributing OTUs responsible for the observed patterns of separation between groups and similarity within groups. Multivariate analyses and nMDS plots were completed using PRIMER-E 7 Ltd. (Clarke and Warwick, 2001).

## Results

The data presented here are based on the final high confidence rarefied (*n* = 831) dataset (Supplementary Data 2). High-confidence non-rarefied data (Supplementary Figure 2A) provided comparable results (data not shown). In total, five adults and their respective bundles as well as a total of nineteen 2-day-old larvae (L2), eighteen 4-day-old larvae (L4), eighteen newly settled spat (Spat), and nineteen 6-month-old juveniles (Juvenile) were successfully sequenced at the 16S rRNA gene (Supplementary Data 2 and Supplementary Figures 2B, 3).

### Source of Microbial Symbionts in *Acropora digitifera* Early Life Stages

**FIGURE 2:**
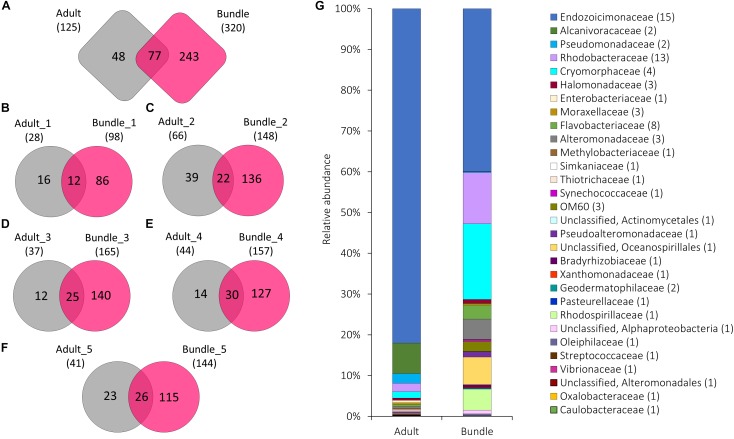
The number of OTUs unique to or shared between **(A)** all five maternal coral colonies (adult) and the released egg-sperm bundles (bundle), and between each individual coral colony and resulting bundles **(B–F)**. The total number of OTUs associated with each group is represented in parentheses. Relative abundance of the 90 bacterial OTUs shared among adult colonies and egg-sperm bundles **(G)** (represented in **A**). Sequences were classified at family level where possible, with unclassified sequences indicated at higher taxonomic resolution. Numbers in parenthesis demark the number of different OTUs within the respective families/taxa.

**FIGURE 3:**
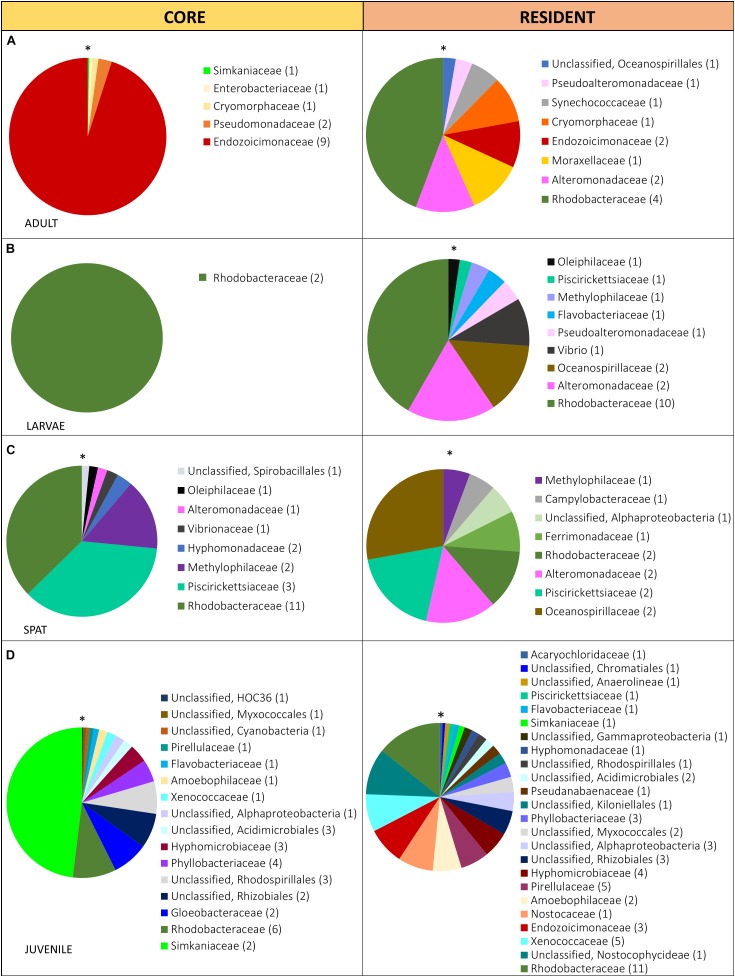
Relative abundance of the bacterial taxa within the core (present in 80% of the samples) and the resident (present in 50% of the samples) microbiome in: **(A)** Adult, adult colonies, **(B)** Larvae, 2- and 4-day-old larvae; **(C)** Spat, newly settled spat; and **(D)** Juvenile, 6-month-old juveniles. Sequences were classified at family level where possible, with unclassified species indicated at higher taxonomic resolution. Families within each pie chart are organized from the lowest (*) to the highest abundance (clockwise direction).

Adult coral colonies hosted 125 bacterial OTUs. Of these, 15 belonged to the Endozoicimonaceae and contributed 76.61% of the mean relative abundance of sequence reads. Other taxa such as Alcanivoracaceae, Trueperaceae, and Streptococcaceae showed mean relative abundances between 7.20 and 0.024% (Supplementary Data 2). A subset of bacterial symbionts were present in both adult coral colonies and egg-sperm bundles (Figure 2A–G). While specific OTUs found in both adult colony and corresponding bundles were variable (Figure 2B–F and Supplementary Figure 4), taxa such as Cryomorphaceae, and Endozoicimonaceae were found in all adults and bundles (Figure 1G and Supplementary Figure 4), and Rhodobacteraceae were found in four out of five adults and associated bundles (Supplementary Figures 4B–E). Of the 15 OTUs belonging to the Endozoicimonaceae, OTU 4397109 was reported among all adult coral colonies (relative abundance between 28.28 and 77.62%) and 20 out of 22 bundle samples (relative abundance between 2.72 and 94.87%) (Supplementary Figure 5). Bundle samples with a lower abundance of OTU 4397109 were all released by the same coral colony (Adult_1) and were dominated by OTU 3041665 (Supplementary Figure 5). Five OTUs belonging to the family Endozoicimonaceae (OTUs 573686, 221108, 4397109, 831558, and 206565) were identified as part of the core bacterial community of adults (Figure 3A), and were also found within each egg-sperm bundle sample (Figure 2B–F and Supplementary Figure 4). Taxa such as Alteromonadaceae (OTUs 823476) and Pseudomonadaceae (OTU 646549) were found in association with four out of five adult coral colonies and their associated bundles (Supplementary Figures 4B–E).

**FIGURE 4:**
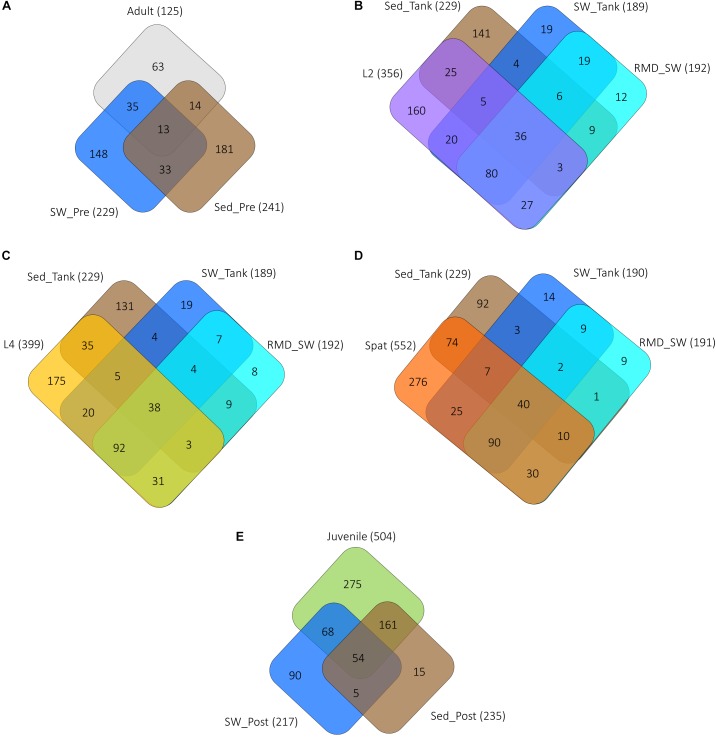
Unique and shared OTUs between the various life history stages and the surrounding environments of seawater and sediment. **(A)** Adult, adult coral colonies; **(B)** L2, 2-day-old larvae; **(C)** L4, 4-day-old larvae; **(D)** Spat, newly settled spat, and **(E)** Juvenile, 6-month-old juveniles. SW, seawater from the reef; SED, sediment from the reef, RMD_SW, reduced microbial density seawater (filtered 0.22 μm). The total number of OTUs associated with each sample type is represented in parentheses. Abbreviations are SW and Sed represent samples taken: “Pre” prior to spawning, “Tank” from the tank experiment, “Post” near juveniles on the reef.

**FIGURE 5:**
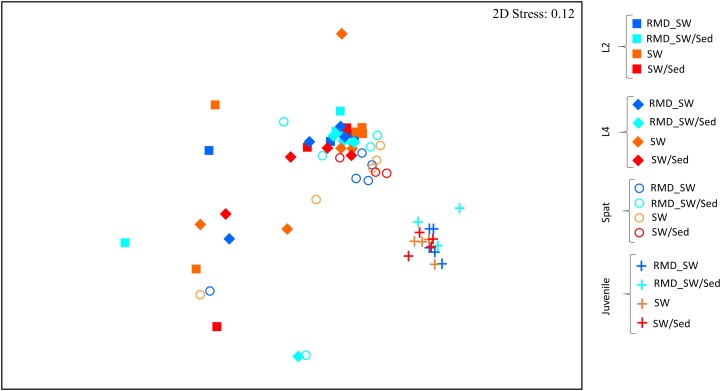
Non-metric multidimensional scaling (nMDS) of entire microbial communities associated with *Acropora digitifera* early life stages and reared among different environmental conditions. Development stages are indicated as: L2, 2-day-old larvae (squares); L4, 4-day-old larvae (diamonds); Spat, newly settled spat (circles); Juvenile, 6-month-old juveniles (cross). While the colors indicated the rearing environment: RMD_SW, reduced microbial cell density seawater (blue); RMD_SW/Sed, reduced microbial cell density seawater and sediment (light blue); SW, seawater (orange); SW/Sed, seawater and sediment (red).

Filtering seawater reduced the bacterial density significantly (one-way ANOVA, *p* < 0.05) (Supplementary Table 1) to approximately 1 × 10^5^ cells/L in comparison to raw seawater with average cell abundances of approximately 1 × 10^8^/L (Supplementary Figure 6), but did not affect the overall composition of the bacterioplankton (PERMANOVA, *p* > 0.05; Supplementary Table 2). Consistent coral bacterial communities were found, regardless of exposure to seawater or sediment bacterial communities (PERMANOVA, *p* = 0.2427; Supplementary Table 3 and Figure 4, 5). Adults and 2-day-old larvae shared more OTUs with the surrounding water column than with sediment (Figure 4A,B) while 4-day-old larvae, spat and juveniles shared more OTUs with the sediment samples (Figure 4C–E). However, the investigation of the bacterial community structure showed that adult coral colonies, L2 and juveniles were more similar to the sediment samples (Supplementary Figures 6A–E,N,O), while L4 and spat had community structure that was similar to both sediment and the water column (Supplementary Figures 6F–M). The number of OTUs shared with both water and sediment increased from the larval stages (Figure 4B,C), to the spat (Figure 4D), and the juveniles (Figure 4E), but then dropped in adult coral colonies (Figure 4A).

### Bacterial Diversity and Dynamics in Early Life Stages of *Acropora digitifera*

**FIGURE 6:**
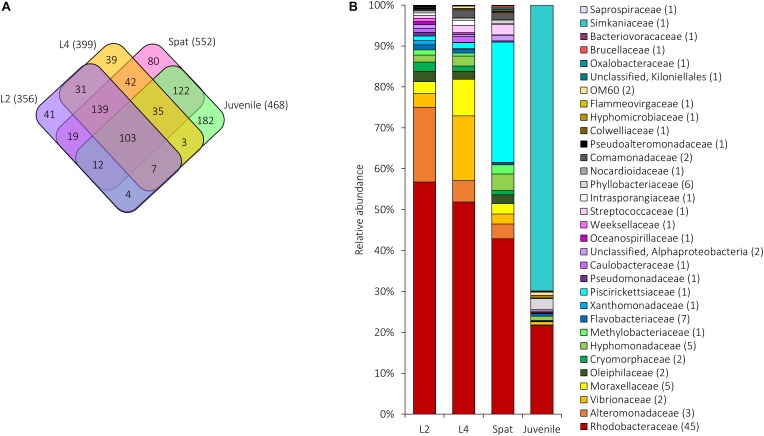
Shared and unique microbial communities associated with *A. digitifera* early life stages: L2, 2-day-old larvae; L4, 4-day-old larvae; Spat, newly settled spat; Juvenile, 6-month-old juveniles. **(A)** The number of OTUs unique to or shared between the development phases. The total number of OTUs associated with each stage is in parentheses. **(B)** Relative abundance of the 109 bacterial OTUs shared among early life stages. Sequences were classified at family level where possible, with unclassified sequences indicated at higher taxonomic resolution. Numbers in parenthesis demark the number of different OTUs within the respective families/taxa.

Each developmental stage of *A. digitifera* had a unique bacterial community (PERMANOVA, *p* < 0.01) (Figure 5 and Supplementary Table 3), with the exception of L2 and L4 (*p* = 0.1773) (Figure 5 and Supplementary Table 3). Bacterial communities also became increasingly complex with early development stages (Figure 6 and Supplementary Table 3).

The microbial communities of 2- and 4-day-old larvae had, respectively, a mean of 76 (±3.07 SE) OTUs from 14 phyla and 75 OTUs (±3.30 SE) from 14 phyla. Both stages were dominated by Proteobacteria (L2 = 78.16%, L4 = 76.37%), Actinobacteria (L2 = 6.94%, L4 = 10.09%), Bacteroidetes (L2 = 10.55%, L4 = 8.19%), and Firmicutes (L2 = 4.14%, L4 = 3.35%). Within spat, 14 phyla were present with an average of 126 ( ± 5.46 SE) OTUs per sample, dominated by Proteobacteria (81.99%), Bacteroidetes (7.66%), Actinobacteria (4.79%), Firmicutes (3.59%), and lower abundance phyla such as *Plancomycetia* (0.11%). Fifteen phyla were identified within the juveniles based on an average of 173 ( ± 4.23 SE) OTUs, and juveniles were dominated by Proteobacteria (48.28%), Chlamydiae (25.26%), Cyanobacteria (14.49%), Bacteroidetes (6.35%), Planctomycetes (2.11%), and Actinobacteria (2.04%) (Supplementary Data 1). Overall, 859 OTUs were found within the early life stages, and significant differences in alpha diversity were identified between early life stages (Kruskal–Wallis *H*-test, *p* < 0.05; Supplementary Table 4A). Both larval stages and spat had a significantly lower number of OTUs and Chao1 index compared to juveniles (see Supplementary Tables 4B,C), while the Shannon index was significantly higher in juveniles compared to the larval stages (Supplementary Table 3D).

All development stages had 103 OTUs in common (Figure 6A). Of these, the relative abundance shifted across development (Figure 6B). Both larvae and spat were dominated by Proteobacteria (L2 = 94.02%, L4 = 94.32%, Spat = 95.40%) which declined to 28.98% in juveniles (Figure 6B). Within the Proteobacteria, members of the Rhodobacteraceae dominated all four stages (L2 = 56.74%, L4 = 51.88%, Spat = 42.87%, Juvenile = 21.63%). Alteromonadaceae reached maximum relative abundance in L2 (18.31%), and then declined in L4 (5.20%), spat (3.60%), and juveniles (0.19%). Xanthomonadaceae slightly increased their highest relative abundance in L2 (1.63%) and then declined in L4 (0.29%) and spat (0.27%), before increasing in juvenile (0.54%). Vibrionaceae (15.86%; OTUs 939811 and 837366) reached their maximum relative abundances within L4. Once larvae settled, the bacterial genus *Methylophaga* (family Piscirickettsiaceae) (OTU 823160) became dominant (29.40%), and then declined (Juveniles = 0.03%). After 6 months exposure to natural environmental conditions, the relative abundance of the genus Simkaniaceae (OTU 217851), increased to 69.85%. Other bacterial taxa such as Bacteroidetes, and Actinobacteria, and Firmicutes were present in lower abundance (0.03–4.24%) across all development stages (Figure 6B).

Bacterial community structure within both larval stages was highly dissimilar to spat [average dissimilarity of 79.76% (L2 vs. Spat) and 78.76% (L4 vs. Spat)]. Taxa such as Piscirickettsiaceae genus *Methylophaga* (OTUs 823160 and 278985) and Methylophilaceae genus *Methylotenera* (OTU 1091363) were predominantly associated with spat rather than larvae. Microbial communities within both larval stages were also dissimilar to juveniles [average dissimilarity = 92.68% (L2 vs. Juvenile) and 92.20% (L4 vs. Juveniles)], predominantly driven by the increased relative abundance of the Chlamydiiae-like OTU 217851 (family Simkaniaceae). Differences between microbial communities of newly settled spat and juveniles (average dissimilarity of 86.98%) were driven by the dominance of the family Simkaniaceae in juveniles, and the genus *Methylophaga* in spat (SIMPER analysis; see Supplementary Table 5).

**FIGURE 7:**
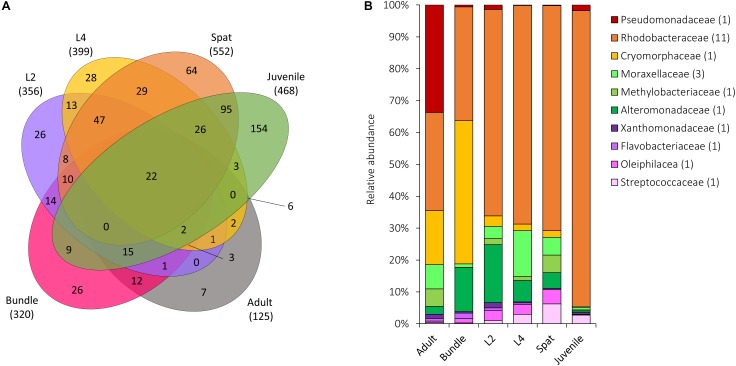
Microbial communities shared across all life history stages of *Acropora digitifera*. **(A)** The number of OTUs unique to or shared between stages, with the total number of OTUs associated with each group represented in parentheses. **(B)** Relative abundance of the 28 bacterial OTUs shared among all generations. Sequences were classified at a family level where possible, with unclassified species indicated at higher taxonomic resolution. Numbers in parenthesis demark the number of different OTUs within the respective families. Adult, adult colonies; Bundle, egg-sperm bundles; L2, 2-day-old larvae; L4, 4-day-old larvae; Spat, newly settled spat; Juvenile, 6-month-old juveniles.

In total, 898 OTUs occurred across coral generations and 22 occurred consistently within all stages (Figure 7A). Rhodobacteraceae were found within each generation and included genera such as *Loktanella* and *Thalassobius* (Figure 6B). Among the OTUs that were shared across all generations (Figure 7B) the Pseudomonadaceae species *Pseudomonas veronii* (OTU 646549) showed a decline in relative abundance from adults to early life history stages. Larvae were characterized by an increased abundance of Alteromonadaceae belonging to the genus *Alteromonas* and to the family Xanthomonadaceae (L2; Figure 7B).

### Core and Resident Bacterial Communities Across Generations

The diversity of the core and the resident bacterial communities varied among development stages (Figure 3). In general, Rhodobacteraceae (i.e., genera *Rhodovulum* and *Loktanella*) were identified among both the core and resident bacterial communities. The dominant core members were characterized by: Endozoicimonaceae (adults, Figure 3A), Rhodobacteraceae (Larvae: L2 and L4; Figure 3B) and Piscirickettsiaceae genus *Methylophaga* (spat, Figure 3C), and Simkaniaceae (Juveniles, Figure 3D). The adult core bacteria Endozoicimonaceae (OTU 555869 and OTU 831558) were identified also in the juvenile resident microbiome (Figure 2A,D). During the adult, larval and spat stages the resident microbiome was characterized by the presence of Alteromonadaceae (Figure 2A–C), and adult and larval resident microbiomes also contained Pseudoalteromonadaceae (Figure 2A,B).

## Discussion

Using 16S rDNA amplicon data, we provide evidence of both vertical and horizontal transmission modes in *Acropora digitifera* and thus suggest that the mechanisms by which bacteria are acquired in corals are more variable (Leite et al., 2017) than previously expected. Some members of the bacterial community within *A. digitifera* displayed a ubiquitous presence in all developmental stages including gametes, yet the core and resident bacterial communities were variable across the host generations. Altogether, this is likely reflective of stage-specific metabolic requirements of the coral host and associated symbionts. Larvae and juveniles harbored more diverse and flexible bacterial communities than adults, supporting the presence of an initial phase represented by a highly diverse and variable bacterial community, followed by a phase of selection (winnowing) and decreased diversity toward the establishment of the final adult consortium (Epstein et al., 2019; Littman et al., 2009a,b). Winnowing processes are known during the establishment host-Symbiodiniaceae partnerships in early life stages of broadcast spawning corals (Abrego et al., 2009; Cumbo et al., 2013), as well as in other holobiont models such as *Hydra* (Franzenburg et al., 2013) and the bobtail squid (Nyholm and McFall-Ngai, 2004). This mechanism of “selective colonization” is likely to be controlled by the host and/or other symbionts (i.e., Symbiodiniaceae or bacteria) for maximum benefit to the physiological requirements specific to each developmental stage and environmental conditions (Little et al., 2004; Nyholm and McFall-Ngai, 2004; Nissimov et al., 2009).

This study demonstrates that *A. digitifera* has the ability to exploit various strategies for the acquisition of bacterial symbionts, indicating flexibility in the mechanisms employed during establishment of symbiotic relationships. The majority of broadcast spawning corals acquire their bacterial symbionts from the surrounding environment as larvae or during the post-settlement and metamorphosis as a juvenile (Weis et al., 2001; Knowlton and Rohwer, 2003; Apprill et al., 2009; Sharp et al., 2010). However, there is some evidence of vertical transmission of Symbiodiniaceae and bacteria from adult broadcast corals to eggs released during spawning (Padilla-Gamiño et al., 2011; Leite et al., 2017). Mixed-mode strategies are common in nature (Russell, 2019) and have been documented in various organisms such as the brooding coral *Seriatopora hystrix* (Quigley et al., 2016) and *Pocillopora damicronis* (Epstein et al., 2019), marine sponges (Schmitt et al., 2008; Webster et al., 2010), solemyid bivalves (Russell et al., 2017), and humans (as reviewed in Ebert, 2013). Mixed strategies combine the best of the horizontal and vertical transmission modes and play an important role in the evolution and ecology of host-symbiont relationships (Bayer et al., 2013; Ebert, 2013; Russell et al., 2017). For instance, through vertical transmission the offspring benefit by not having to “search” for symbionts and transmission of beneficial partners (Byler et al., 2013). However, in environmental conditions different from those of the parents, this may confer a disadvantage if the transmitted symbionts are not optimal. In horizontal acquisition, the host can take up genetically diverse symbionts well suited to a specific environment but they may lack beneficial symbionts inherited by the parental colony (Byler et al., 2013). Therefore, corals showing both strategies are likely to be physiologically and evolutionarily advantaged compared to those strictly relying on one or the other.

We report the presence of bacterial taxa that were found in association with specific life stages, and ubiquitous taxa with either stable or fluctuating relative abundances, suggesting that proliferation of some microbial partners was promoted while others were suppressed (Little et al., 2004). These processes could represent a mechanism of “competitive exclusion” were microbes compete for the resources available (Hibbing et al., 2010). However, we hypothesize that changes in the relative abundance of bacterial phenotypes reflect variations in metabolic requirements at specific life stages. Clostridiaceae were reported within 4-day-old larvae and newly settled spat. Members of the Clostridiaceae, as well as Rhodobacteraceae and the genus *Vibrio*, contribute to carbon and/or nitrogen availability through the degradation of chitin produced by symbiotic zooxanthellae (Wainwright, 1963; Neulinger et al., 2008; Bourne et al., 2016). Larvae were dominated by the diazotrophic bacteria *Alteromonas* and *Vibrio*, which are expected to provide an additional source of fixed nitrogen to the host (Lema et al., 2014) and associated Symbiodiniaceae (Olson et al., 2009). Potential beneficial roles of *Alteromonas* include protection against pathogens (Gil-Turnes et al., 1989), and induction of larval settlement (Webster et al., 2004; Ceh et al., 2013). The protective and nutritional properties of these taxa could be fundamental during the first vulnerable life stages, where larvae face a gradual decline in metabolism rate expressed by oxygen consumption (Nakamura et al., 2011), but are still capable of swimming up to 200 days without feeding (Graham et al., 2008).

In the spat and juvenile phases we found the presence and increased relative abundance of bacterial taxa involved in the sulfur cycle (Raina et al., 2010). Specifically, the relative abundance of two common coral-associated dimethylsulfoniopropinate (DMSP) degrading bacteria (Raina et al., 2010; Leite et al., 2018), Desulfovibrionaceae and Desulfobacteraceae, increased in spat and juveniles. DMSP is likely to be synthesized by both the host and Symbiodiniaceae (Van Alstyne et al., 2006; Raina et al., 2013), and degradation of DMSP produces DMS, acrylate and dimethylsulfoxde (DMSO) which, due to their antioxidant properties, are involved in stress response (Raina et al., 2013, 2016; Deschaseaux et al., 2014) and antimicrobial activities (Slezak et al., 1994; Broadbent and Jones, 2004). Spat were also characterized by the presence of OTUs belonging to dimethylsulfide (DMS) degrading *Methylophaga*, while within juveniles this role may be played by other taxa such as *Vibrio*, *Pseudomonas*, and *Alteromonas* (Raina et al., 2010). Spat were also characterized by an increased relative abundance of a common coral-associated bacteria (Silveira et al., 2017), *Janthinobacterium*, known to produce antifungal metabolites (Brucker et al., 2008). The early establishment of these symbioses is likely to contribute beneficially to biological processes such as growth as well as protection at highly vulnerable life stages.

Spat and juveniles hosted an increased relative abundance of Rhizobiales, and juveniles hosted a higher relative abundance of Simkaniaceae. Members of the genus Simkaniaceae are among the most common bacterial taxa associated with both corals (Bayer et al., 2013; Ogawa, 2014; Wright et al., 2016) and octocorals (van de Water et al., 2018), however, their functional role is unknown. Rhizobiales are nitrogen fixing bacteria frequently reported in early life stages of corals (Sharp et al., 2012; Lema et al., 2014) and may be specifically associated with Symbiodiniaceae (Olson et al., 2009; Ainsworth et al., 2015). This could signify that they provide nutrients to Symbiodiniaceae which are in turn responsible for the production of chitin involved in the formation of the skeleton (Watanabe et al., 2003) which starts developing within the juvenile stage (Wainwright, 1963). The presence of diazotroph bacteria across early life stages highlights the possible functional importance of nitrogen fixing bacteria for the coral holobiont (Lema et al., 2014), due to their ability to provide additional source of fixed nitrogen in oligotrophic waters (Lesser et al., 2004, 2007; Olson et al., 2009; Lema et al., 2012, 2014). Early life stages were all characterized by the ubiquitous presence of members of the family Xanthomonadaceae which are known to promote plant growth and suppress pathogenic symbionts (Li et al., 2015) and establish symbiotic relationships with a wide range of organisms such as beetle larvae (Geib et al., 2009; Rizzi et al., 2013) as well as coral (Polson, 2007; Ceh et al., 2012; Godwin et al., 2012; Beatty et al., 2018).

Taxa such as Cryomorphaceae and the substantially dominant Endozoicimonaceae were ubiquitously found in bundles, while members of the Rhodobacteraceae, Alteromonadaceae, and Pseudomonadaceae were reported in four out of five adult/bundle individuals and across generations. Within the Endozoicimonaceae OTUs identified in adult and associated bundle OTU 4397109 matched in NCBI (100% similarity) with *Endozoicimonas eunicola* associated with the octocorals *Eunicea fusca* and *Plexaura* sp. (Accession number: NR_109684). Therefore, suggesting that vertical transmission ensures the generational transfer of beneficial microbial taxa likely to possess specific mechanisms to promote the coral host growth, development, nutrition and protection.

Here, both seawater and sediment surrounding the coral host were identified as potential reservoirs of microbes acquired throughout coral development. *Methylophaga* (family Piscirickettsiaceae; OTU 823160) and *Pseudomonas* (OTU 646549) were likely acquired during larval stages from sediment and seawater, respectively. Endozoicimonaceae, found in both adults and juveniles, were most likely taken up from the seawater at both larval and post-settlement stages. However, some of the bundles were associated with the same OTUs (i.e., *Pseudomonas* OTU 646549), therefore suggesting that they are also likely to be transmitted by the parental coral colony. The mixed transmission mode documented in this study may represent a functional compromise between horizontal and vertical strategies. Indeed, as suggested by Russell et al. (2017) horizontal transmission improves deleterious mutations and genome stasis through the facilitation of recombination and horizontal gene transfer. Vertical transmission supports the evolution of co-adapted host-symbiont genotypes, but also guarantees the colonization of each generation with symbionts. Thus, by combining both strategies, populations might be able to respond more rapidly to short and long term evolutionary pressures (Kaltz and Koella, 2003; Byler et al., 2013).

The core bacterial community has been proposed to be intimately linked with host requirements, while the variable community is thought to be influenced by surrounding environmental conditions (Hernandez-Agreda et al., 2016). We describe distinct core (>80% of the samples of each life stage) and resident bacterial communities (50–79% of each life stage samples) in the various life history stages, and propose that changes in communities reflect the ability of the host to adapt their microbiome to the distinct metabolic and/or environmental requirements of each stage. We also verified that the resident microbiome was characterized by a higher diversity than the core bacterial communities. The core bacterial communities of adults was dominated by the Endozoicimonaceae, as has previously been reported (Ainsworth et al., 2015; Hernandez-Agreda et al., 2018). Recently members of the Endozoicimonaceae have been described as being able to recognize, translocate, communicate with and modulate the coral host (Ding et al., 2016), as well as contribute to protein provision and cycling of carbohydrates (Neave et al., 2017). Interestingly, Endozoicimonaceae did not appear within the core or resident microbiome of larvae or spat, but subsequently became part of the juvenile resident microbiome. Similarly, members of the Simkaniaceae were dominant taxa within the juvenile core microbiome and part of the adult core microbiome. The simultaneous presence of Endozoicimonaceae and Simkaniaceae (Bayer et al., 2013; Pogoreutz, 2016; Goldsmith et al., 2018) could indicate that they play a critical functional role and/or that there is an interaction between the two. The core microbiome of spat and juveniles was dominated by Rhodobacteraceae, Piscirickettsiaceae, and Simkaniaceae, respectively. The presence of other taxa (i.e., *Inquilinus*), was previously reported as part of the core microbiome in early life stages (Leite et al., 2017) but this is the first report of core/resident bacterial communities changing through development. Temporal dynamicity of the core bacterial communities may enable the identification of consistent members across complex bacterial assemblages which are likely to facilitate the development, functioning and health of the host during each development stage.

While each stage hosted a distinct bacterial community, a notable component of both the core and resident bacterial communities of the various stages was the presence of the Rhodobacteraceae which has previously been identified (Williams et al., 2015) as ubiquitously distributed across coral life stages. The consistent presence of Rhodobacteraceae and *Pseudomonas* had been identified previously within the core bacterial communities of coral mucus within a broadcast spawning species (Leite et al., 2018), and were vertically transmitted (Leite et al., 2017). These studies and ours provide mounting evidence that components of the core bacterial communities can potentially be transmitted vertically across generations. The importance of Rhodobacteraceae might be due to their ability to obtain energy via sulfur oxidation, their ability to scavenge small amounts of organic material as a source of carbon (Neulinger et al., 2008), and their tolerance of anaerobic conditions such as those found in the mucus of tropical corals (Kellogg, 2004), and in polyps after feeding (Neulinger et al., 2008). We suggest that the core and resident coral bacterial communities are likely determined by the host, and possibly by the interaction with symbionts. However, the specific mechanisms that facilitate this selective process are still relatively unknown and require further investigation. It is noteworthy that some bacterial taxa found within these putatively key communities (i.e., Alteromonadaceae, Vibrionaceae, Simkaniaceae, Desulfobacteraceae, and Desulfovibrionaceae) include species commonly found within corals (Bernasconi et al., 2018) that can be beneficial as well as responsible for the outbreak of coral diseases (Bourne et al., 2009). As our specimens were healthy, these members may be considered part of the natural coral microbiome and opportunistic pathogens.

## Conclusion

Our study shows that the mixed mode acquisition and subsequent winnowing of symbionts are likely to represent fundamental mechanisms in the ecology and evolution of corals. Particularly, in changing environmental conditions, mixed mode acquisition may represent a mechanism for the formation of new beneficial symbioses (Byler et al., 2013) and of new interactions with other symbionts (i.e., Symbiodiniaceae) within the coral host. Future investigations must focus on mechanisms behind the specific uptake of the microbial communities, including the roles played by other symbionts. As the condition of reefs decline worldwide (Paredes et al., 2003; Birkeland, 2019) coral taxa need to adapt to rapidly changing environmental conditions or risk extinction. We advocate that a better understanding of how microbes are acquired and utilized by corals is necessary to develop a more complete picture of coral resilience and adaptation.

## Data Availability

The datasets generated for this study can be accessed from the NCBI Sequence Read Archive. The BioProject ID is PRJNA526947, accession numbers SAMN11097120 – SAMN11097248 and SAMN11603544-SAMN11603546.

## Ethics Statement

Permission to conduct the fieldwork and collect coral samples was granted by the WA Department of Parks and Wildlife (permit#SF010989) and WA Department of Fisheries (permit#2895). No special animal ethics approval was needed.

## Author Contributions

RB, MS, AK, and MH designed the experiment and interpreted the results. RB and MH collected the samples, ran the experiment, and wrote the manuscript. RB analyzed the data. MS and MB assisted RB with laboratory work and data analyses and AP with the bioinformatics analyses. MS, AK, AP, and MB edited the manuscript.

## Conflict of Interest Statement

The authors declare that the research was conducted in the absence of any commercial or financial relationships that could be construed as a potential conflict of interest.

## Footnotes

1 https://www.geneious.com
