# Supplementary material for: Establishment of Coral-Bacteria Symbioses Reveal Changes in the Core Bacterial Community With Host Ontogeny
Source: Front Microbiol. 2019 Jul 9;10:1529. doi: 10.3389/fmicb.2019.01529 (PMC6629827; doi:10.3389/fmicb.2019.01529)
Supplement: Supplementary file 1 [file Data_Sheet_1.docx]

**Supplementary Figures**

**
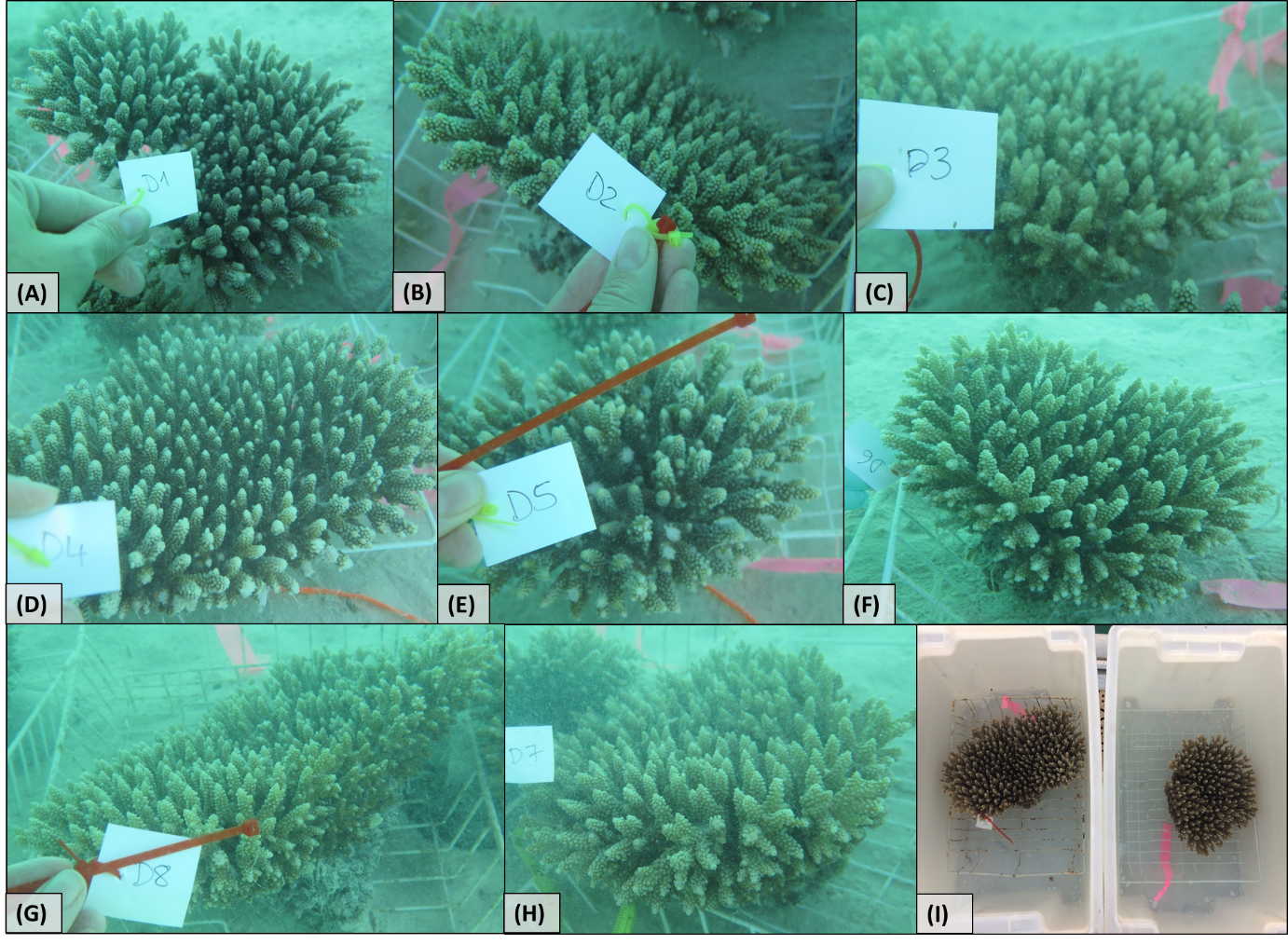
**

**Supplementary Figure 1. (A-H)** Eight healthy adult colonies of *Acropora digitifera* were collected from Ningaloo Reef in Coral Bay (Western Australia) and transported to the Coral Bay jetty a few hours prior to spawning, colonies were placed into individual 60 L tubs containing seawater **(I)**.

**
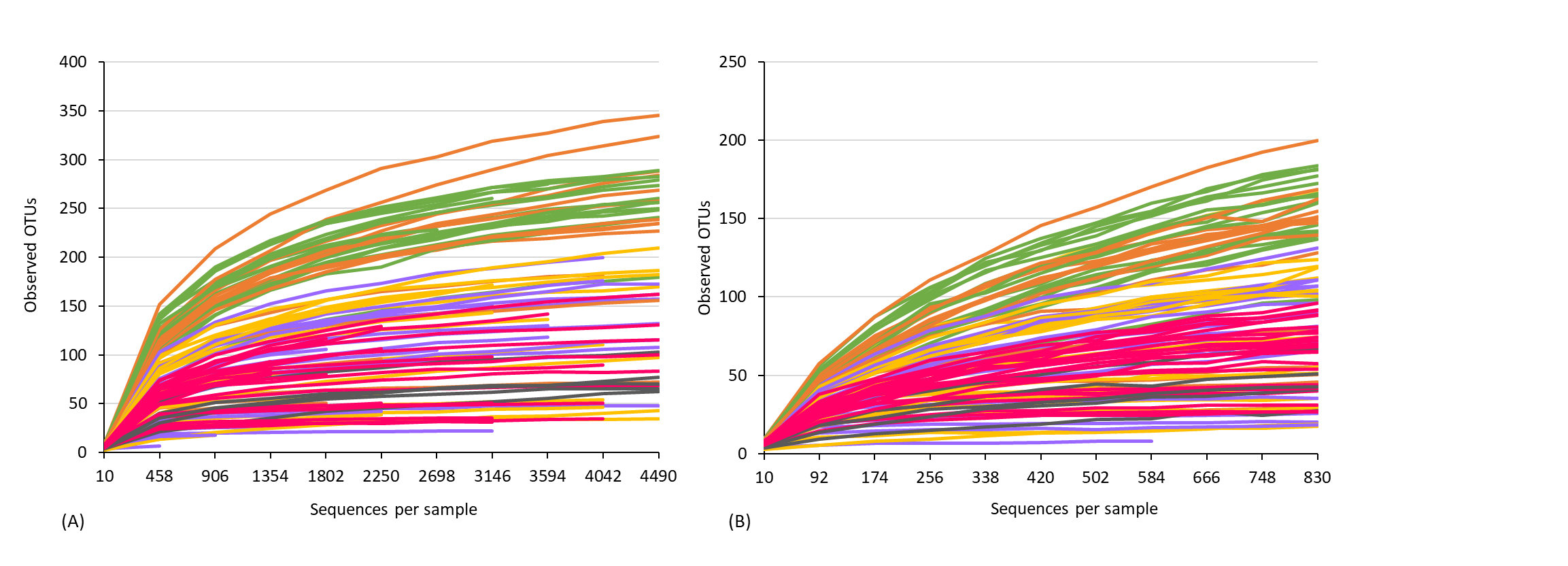
**

**Supplementary Figure 2.** Rarefaction curves of OTUs for the bacterial communities from coral development stages (adult, bundle, 2 and 4-day-old larvae, spat and juvenile), and the surrounding environment (seawater, reduced microbial cell density seawater and sediment). The rarefaction curves tend to approach the saturation plateau on both **(A)** non-rarefied, and **(B)** rarefied data.


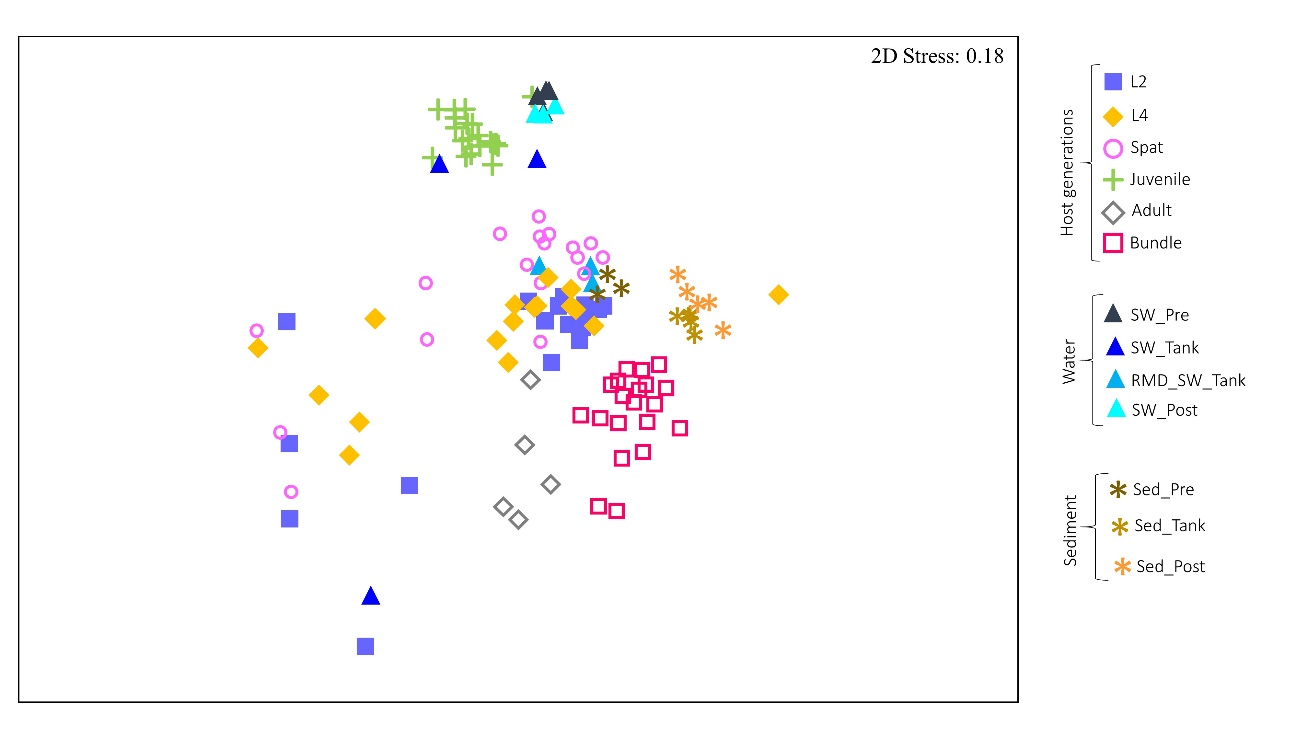


**Supplementary Figure 3.** Non-metric multidimensional scaling (nMDS) of entire bacterial communities associated with all *A. digitifera* generations and all environmental samples. Development stages are indicated as: L2, 2-day-old larvae (filled squares); L4, 4-day-old larvae (filled diamonds); Spat, newly settled spat (circles); Juvenile, 6-months-old juvenile (cross); Adult, adult coral colonies (empty diamonds); Bundle, egg-sperm bundles (empty squares). While environmental samples are indicated as: SW_Pre, seawater collected before the spawning event, (dark blue triangles); SW_Tank, seawater added to the treatments (bright blue triangles); RMD_SW, reduced microbial cell density seawater (light blue triangles); SW_Post, seawater collected after the spawning event (turquoise triangles); Sed_Pre, sediment collected before the spawning event (dark brown asterisks); Sed_Tank, sediment added to the treatments (brown asterisks); Sed_Post, sediment collected after the spawning event (light brown asterisks).

**
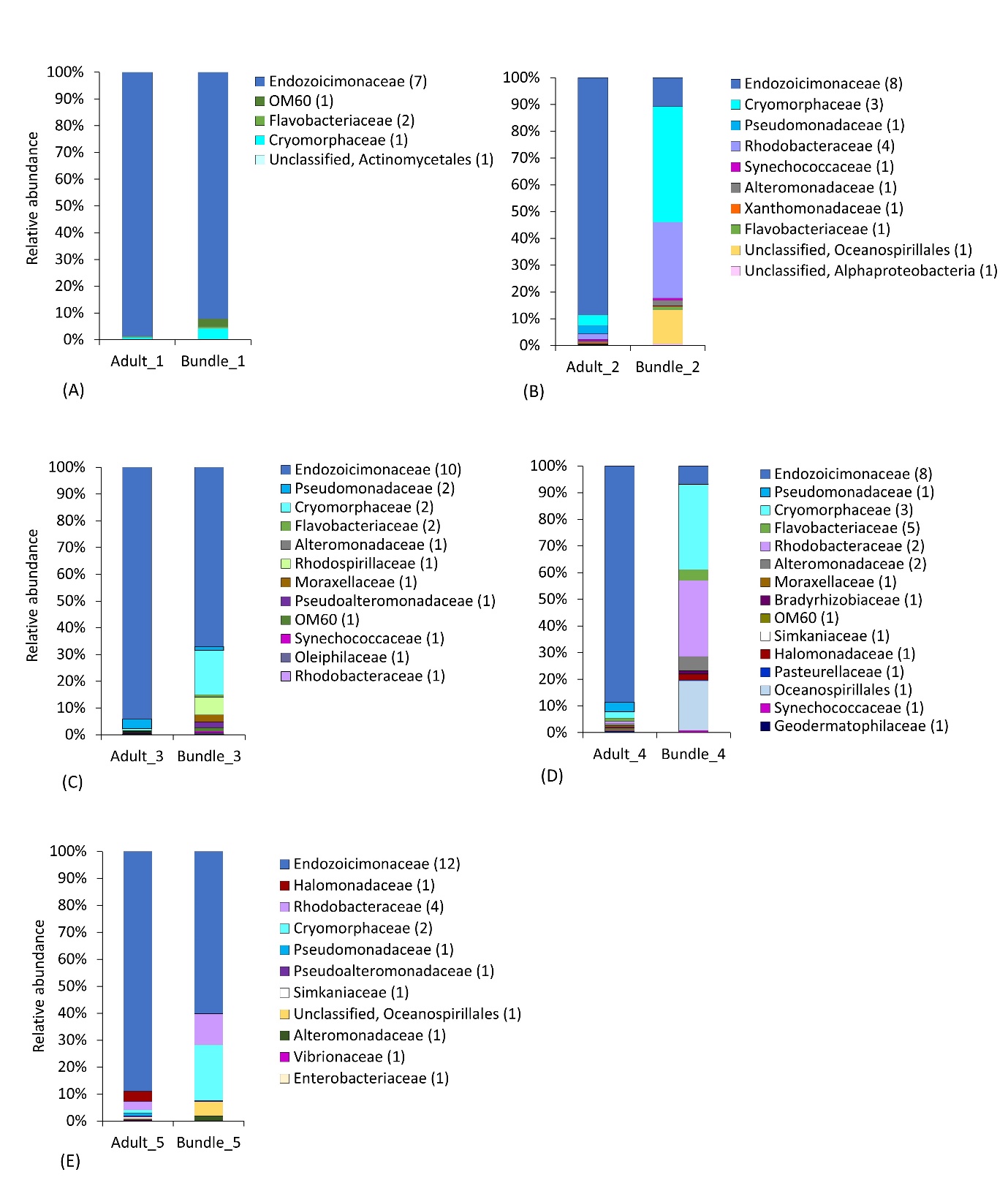
**

**Supplementary Figure 4.** Relative abundance of the bacterial OTUs shared among each adult coral colony and their respective egg-sperm bundles: **(A)** Adult_1 and Bundle_1; **(B)** Adult_2 and Bundle_2; **(C)** Adult_3 and Bundle_3; **(D)** Adult_4 and Bundle_4; **(E)** Adult_5 and Bundle_5. Sequences were classified at a Family level where possible, with unclassified species indicated at higher taxonomic resolution. Numbers in parenthesis demark the number of different OTUs within the respective families.


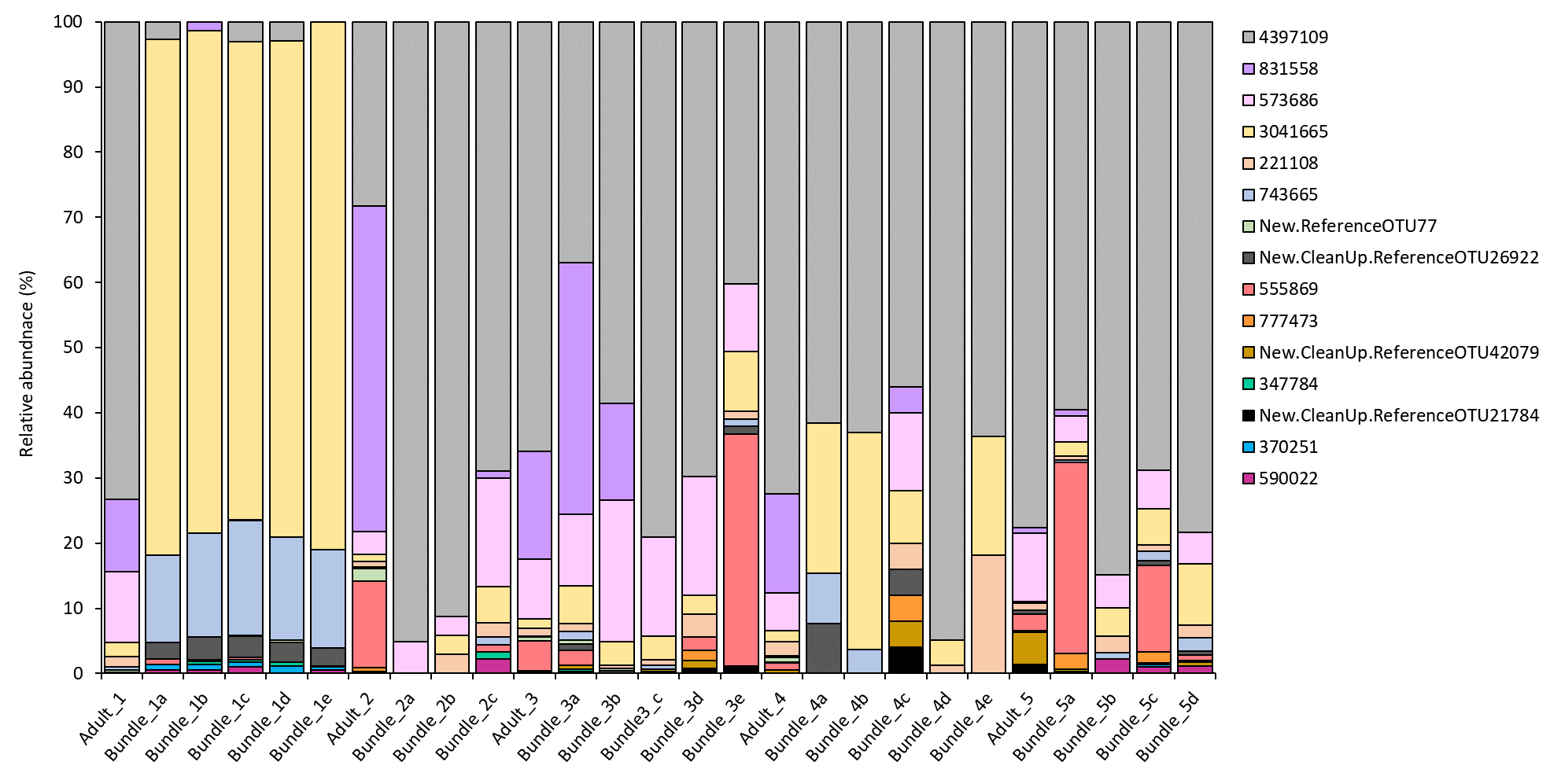


**Supplementary Figure 5.** Relative abundance of the Endozoicimonaceae OTUs shared among each adult coral colony and their respective egg-sperm bundles.

**
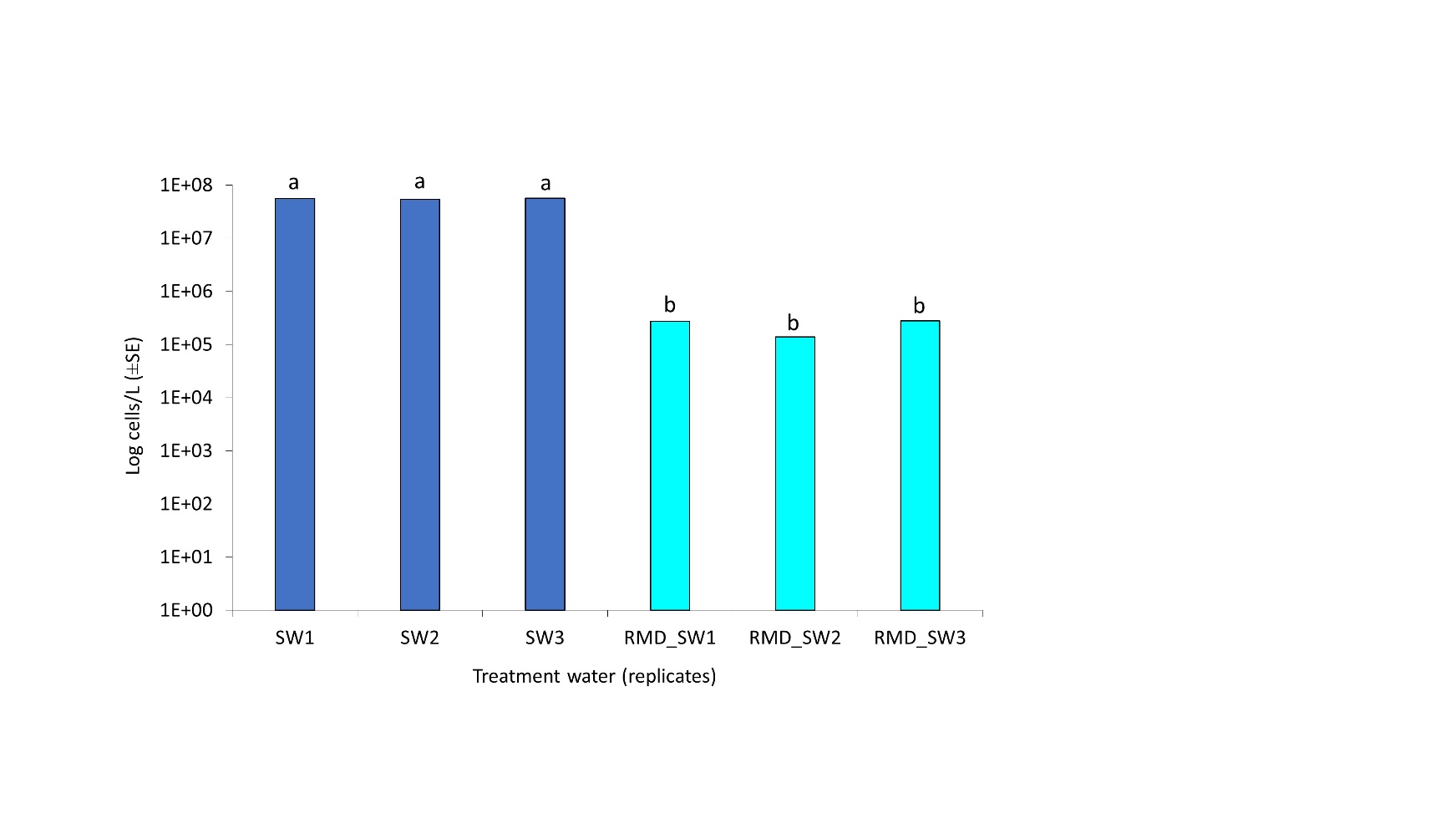
**

**Supplementary Figure 6.** Measure of bacterial abundance (±SE) by SYBR Gold count in seawater (SW) and reduced microbial cell density seawater (RMD_SW). Samples that share the same letter are not significantly different form one other (one-way ANOVA, p<0.05). Standard Error bars (SE) values showed a variability of the data too small to be visible in the bar graph.

**
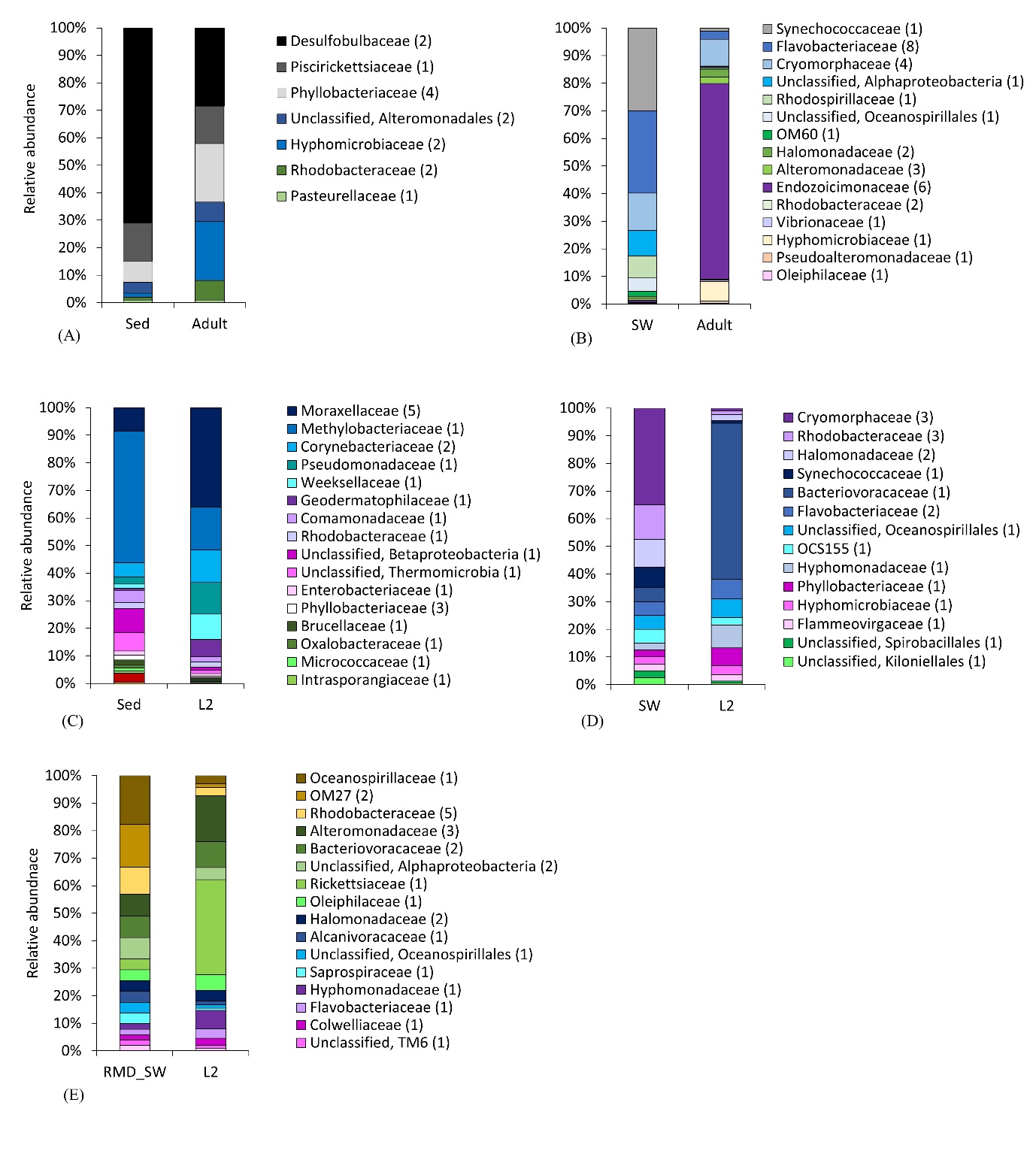
**

**
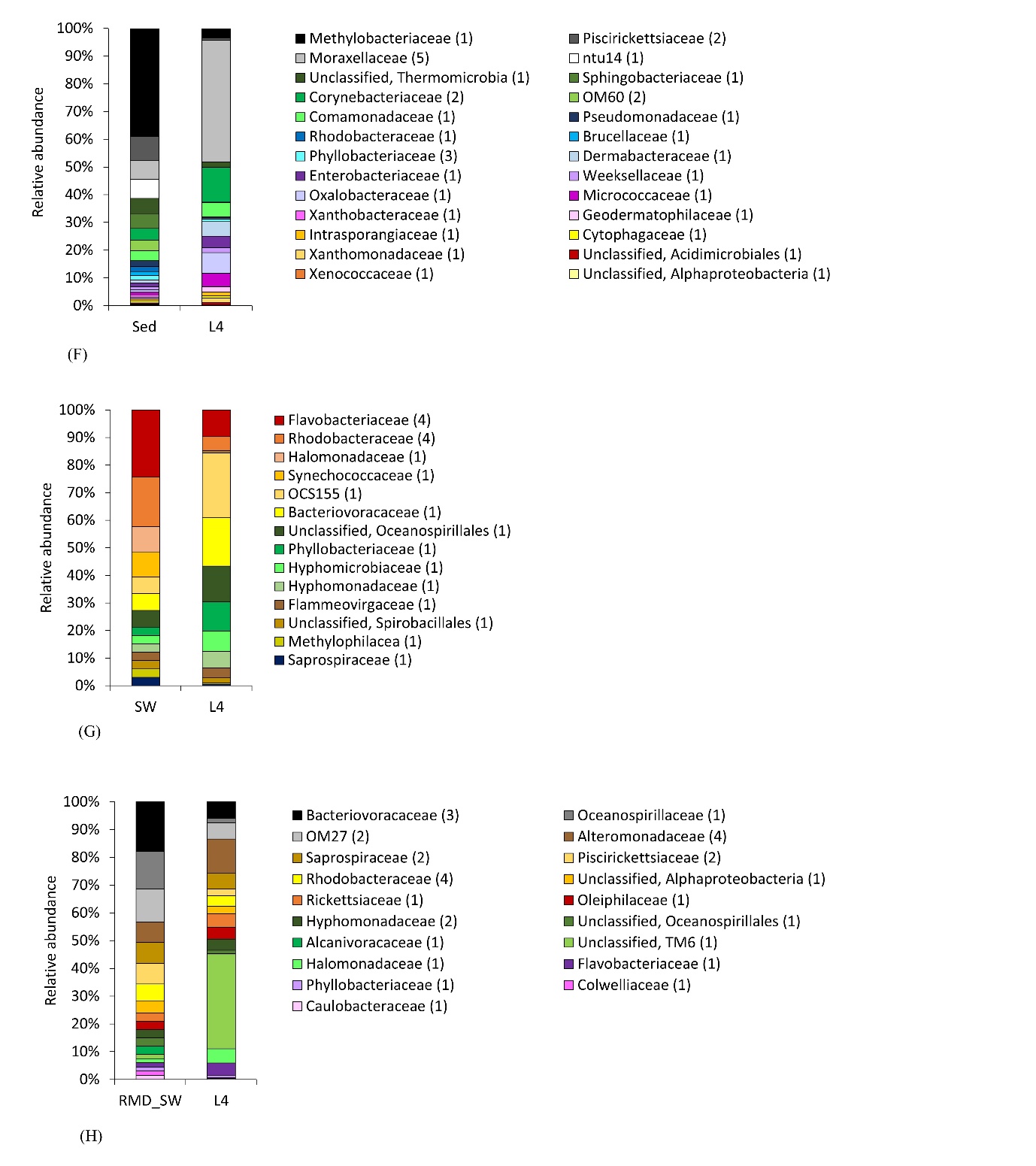
**

**
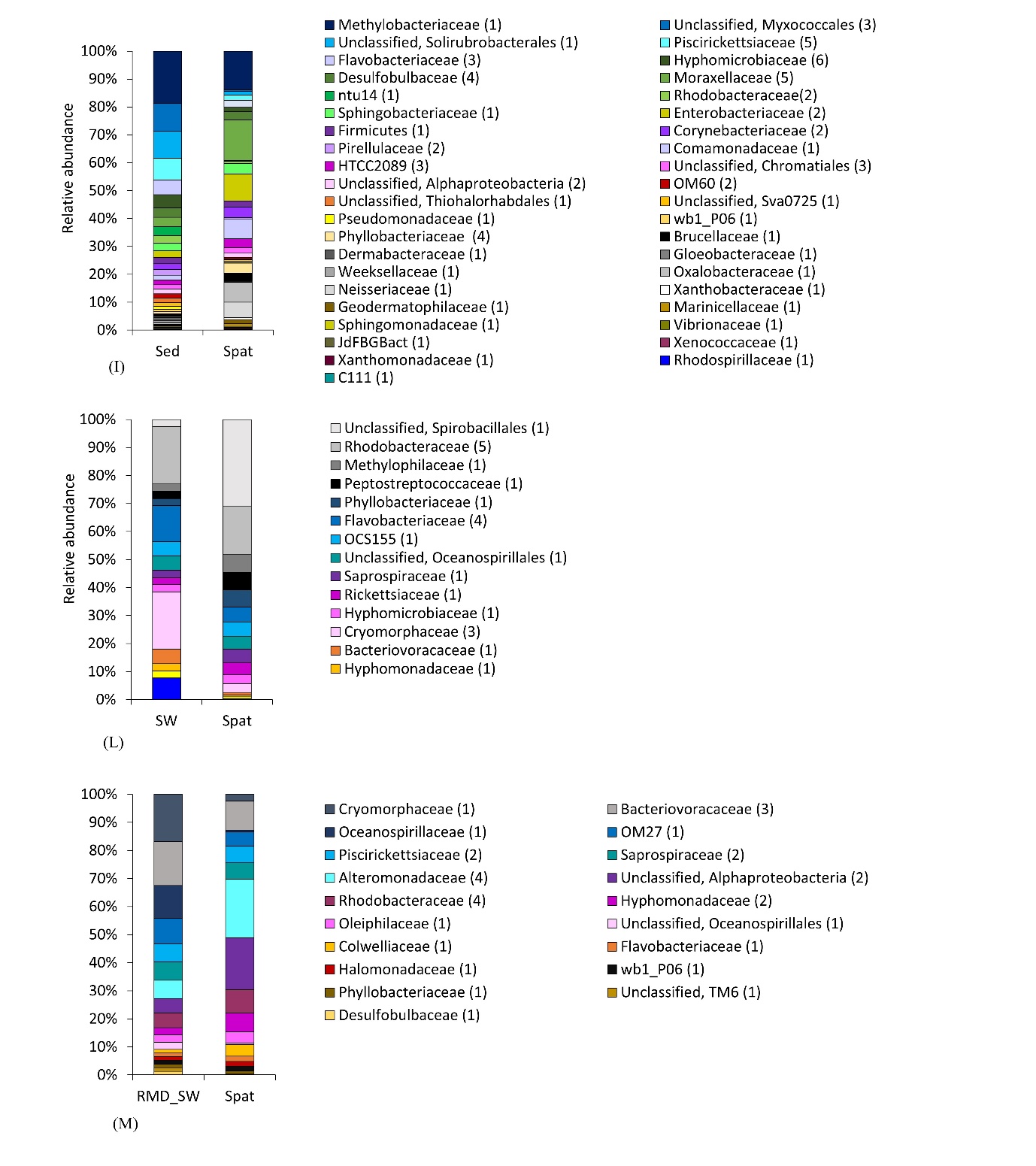
**

**
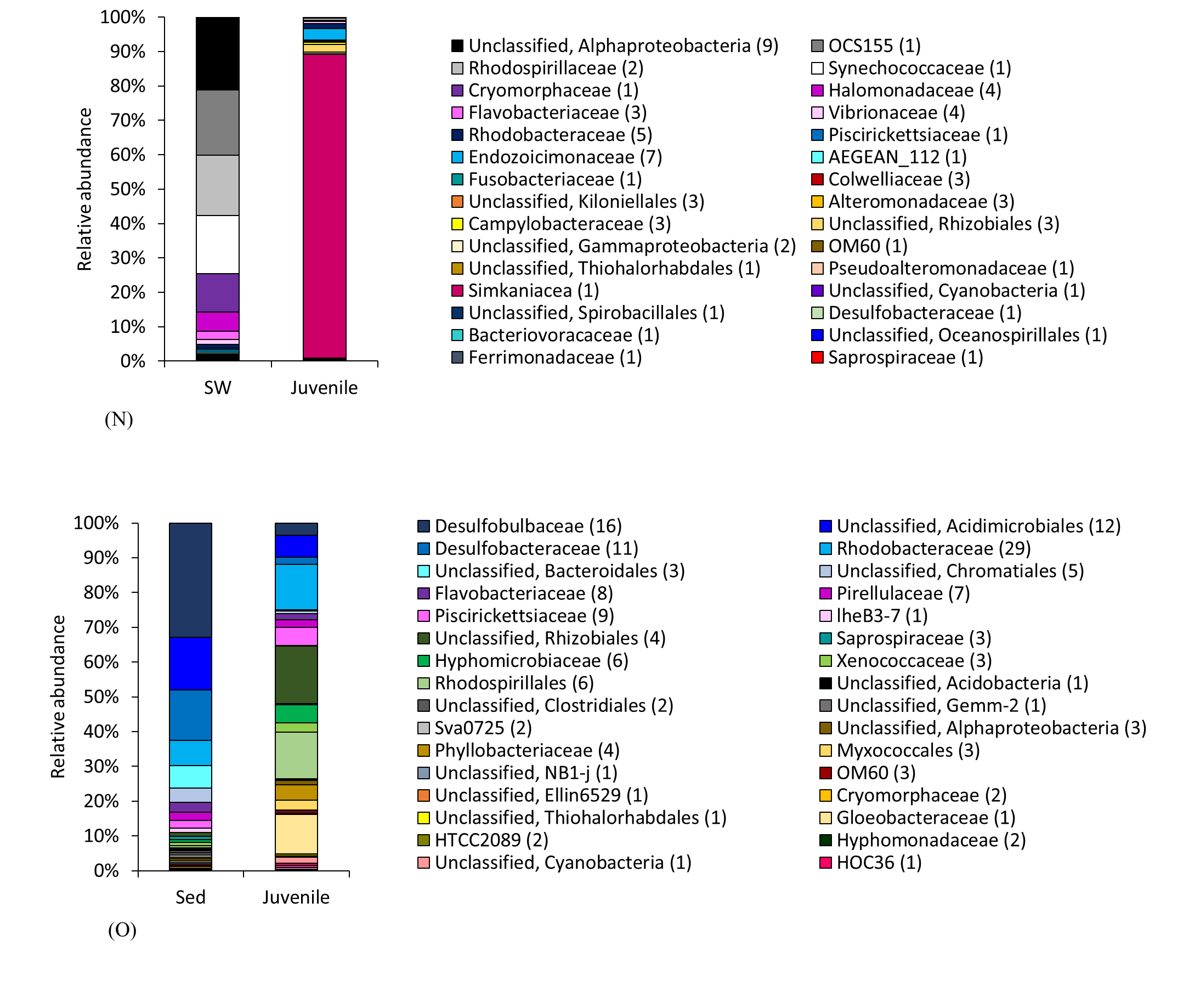
**

**Supplementary Figure 7.** Relative abundance of the bacterial OTUs shared between adult coral colonies and **(A)** sediment (Sed), or **(B)** seawater (SW). Between 2-day-old larvae (L2) and **(C)** sediment (Sed), or **(D)** seawater (SW) or **(E)** reduced microbial cell density seawater (RMD_SW). Between 4-day-old larvae (L4) and **(F)** sediment (Sed) or **(G)** seawater (SW), or **(H)** reduced microbial cell density seawater (RMD_SW). Between newly settle spat (spat) and **(I)** sediment (Sed), or **(L)** seawater (SW); **(M)** reduced microbial cell density seawater (RMD_SW). Between 6-months-old juvenile (Juvenile) and **(N)** sediment (Sed) or **(O)** seawater (SW). Sequences were classified at Family level where possible, with unclassified sequences indicated at higher taxonomic resolution. Numbers in parenthesis demark the number of different OTUs within the respective families/taxa.
