# Supplementary material for: Establishment of Coral-Bacteria Symbioses Reveal Changes in the Core Bacterial Community With Host Ontogeny
Source: Front Microbiol. 2019 Jul 9;10:1529. doi: 10.3389/fmicb.2019.01529 (PMC6629827; doi:10.3389/fmicb.2019.01529)
Supplement: Supplementary file 2 [file Data_Sheet_2.docx]

**Supplementary Tables**

**Supplementary Table 1.** Outputs of one-way ANOVA for changes in microbial density between seawater and reduced microbial density seawater samples. Statistically significant factors (p <0.05) are indicated in bold, with the p value also italicised. p= p-value.

| Main test (one-way ANOVA) | df | F | p |
| --- | --- | --- | --- |
| Between Groups | **1** | **41.316** | ***<0.005*** |
| Within Groups | 86 |  |  |

**Supplementary Table 2.** Outputs of PERMANOVA main test comparing the bacterial community structure between seawater and reduced microbial density seawater. df= degree of freedom, Pseudo-F= permuted F-value, p= p-value.

| Main test (1 factor PERMANOVA) | df | Pseudo-F | Permutations | p |
| --- | --- | --- | --- | --- |
| Water type (Wa) | 1 | 1.1186 | 9999 | 0.4034 |
| Res | 4 |  |  |  |
| Total | 5 |  |  |  |

**Supplementary Table 3.** Outputs of PERMANOVA main test and pairwise tests comparing the bacterial community structure across treatments and development stages. Statistically significant treatments (p <0.01) are indicated in bold, with the p value also italicised. To assess which development stages were significantly different, pair-wise tests were performed and these results are displayed in Figure 5 in the main document. df= degree of freedom, Pseudo-F= permuted F-value, p= p-value.

| Main test (2 factor PERMANOVA) | df | Pseudo-F | Permutations | p |
| --- | --- | --- | --- | --- |
| Development (De) | **3** | **9.5211** | **9862** | ***0.0001*** |
| Treatment (Tr) | 3 | 1.1405 | 9887 | 0.2427 |
| De x Tr | 9 | 0.80096 | 9802 | 0.9366 |

| Pairwise test for De | t | p |
| --- | --- | --- |
| L2, L4 | 1.1134 | 0.1773 |
| L2, Spat | **2.124** | ***0.0001*** |
| L2, Juvenile | **4.6762** | ***0.0001*** |
| L4, Spat | **1.6248** | ***0.0033*** |
| L4, Juvenile | **4.2067** | ***0.0001*** |
| Spat, Juvenile | **4.0945** | ***0.0001*** |

**Supplementary Table 4. (A)** Richness and alpha diversity of the bacterial communities associated with *A. digitifera* early life stages. Outputs of Kruskal-Wallis H test for **(B)** OTUs richness; **(C)** Shannon’ index; and **(D)** Chao1 index. Statistically significant factors (p<0.05) are indicated in bold, with the p value also italicised. To assess which treatment were significantly different, Dunn’s post hoc test were performed and these results are displayed on the main document. df= degree of freedom, p= p-value, adj. p = significance value has been adjusted by the Bonferroni correction for multiple tests.

| Development | OTUs (±SE) | Shannon’s H (±SE) | Chao1 (±SE) |
| --- | --- | --- | --- |
| L2 | 53.14 (2.14) | 4.47 (0.06) | 75.91 (3.07) |
| L4 | 51.33 (2.23) | 4.17 (0.08) | 74.69 (3.30) |
| Spat | 80.19 (3.39) | 4.98 (0.064) | 126.29 (5.46) |
| Juvenile  **(A)** | 103.14 (3.29) | 4.99 (0.69) | 172.51 (4.23) |

| Development | Mean Rank |
| --- | --- |
| L2 | 325.81 |
| L4 | 314.02 |
| Spat | 460.89 |
| Juvenile | 573.08 |

| Pairwise comparisons Dunn’s test – OTU richness | | | |
| --- | --- | --- | --- |
| (I) Development | (J) Development | p | adj. p |
| L2 | L4 | 0.613 | 1.000 |
|  | **Spat** | ***<0.001*** | ***<0.001*** |
|  | **Juvenile** | ***<0.001*** | ***<0.001*** |
| L4 | **Spat** | ***<0.001*** | ***<0.001*** |
|  | **Juvenile** | ***<0.001*** | ***<0.001*** |
| Spat | **Juvenile** | ***<0.001*** | ***<0.001*** |

|  | OTUs |
| --- | --- |
| *Kruskal-Wallis H* | 163.995 |
| df | 3 |
| *Sig.* | ***<0.001*** |

**(B)**

| Development | Mean Rank |
| --- | --- |
| L2 | 360.28 |
| L4 | 321.77 |
| Spat | 490.79 |
| Juvenile | 501.22 |

| Pairwise comparisons Dunn’s test – Shannon’s index | | | |
| --- | --- | --- | --- |
| (I) Development | (J) Development | p | adj. p |
| L2 | L4 | 0.099 | 0.592 |
|  | **Spat** | ***<0.001*** | ***<0.001*** |
|  | **Juvenile** | ***<0.001*** | ***<0.001*** |
| L4 | **Spat** | ***<0.001*** | ***<0.001*** |
|  | **Juvenile** | ***<0.001*** | ***<0.001*** |
| Spat | Juvenile | 0.662 | 1.000 |

|  | OTUs |
| --- | --- |
| *Kruskal-Wallis H* | 89.029 |
| df | 3 |
| *Sig.* | ***<0.001*** |

**(C)**

| Development | Mean Rank |
| --- | --- |
| L2 | 297.66 |
| L4 | 297.17 |
| Spat | 464.90 |
| Juvenile | 615.36 |

| Pairwise comparisons Dunn’s test – Chao1 index | | | |
| --- | --- | --- | --- |
| (I) Development | (J) Development | p | adj. p |
| L2 | L4 | 0.983 | 1.000 |
|  | **Spat** | ***<0.001*** | ***<0.001*** |
|  | **Juvenile** | ***<0.001*** | ***<0.001*** |
| L4 | **Spat** | ***<0.001*** | ***<0.001*** |
|  | **Juvenile** | ***<0.001*** | ***<0.001*** |
| Spat | **Juvenile** | ***0.003*** | ***0.031*** |

|  | OTUs |
| --- | --- |
| *Kruskal-Wallis H* | 255.109 |
| df | 3 |
| *Sig.* | ***<0.001*** |

**(D)**

**Supplementary Table 5.** SIMPER outputs showing OTUs that contribute the most to average dissimilarity between development stages: L2, 2-day-old larvae; L4, 4-day-old larvae; Spat, newly settled spat; Juvenile, 6-months-old juvenile. OTUs that contribute cumulatively to 20% of the dissimilarity are shown. OTUs have been classified to Family level where possible, with Unclassified species indicated at higher taxonomic resolution. Av. Abund= average abundance; Av. Diss=average dissimilarity, Diss/SD=average contribution/standard deviation, % Contrib=% OTU contribution, Cum. %= cumulation of OTU contribution.

*Average dissimilarity between L2 and spat: 79.76%*

| OTU | Av. Abund (L2) | Av. Abund (Spat) | Av. Diss | Diss /SD | % Contrib | Cum. % | Taxonomy |
| --- | --- | --- | --- | --- | --- | --- | --- |
| 823160 | 0.54 | 2.66 | 0.93 | 1.38 | 1.17 | 1.17 | *Piscirickettsiaceae* |
| 1091363 | 0.26 | 2.06 | 0.71 | 1.60 | 0.88 | 2.05 | *Methylophilaceae* |
| 248432 | 2.13 | 0.93 | 0.65 | 1.22 | 0.81 | 2.86 | *Oceanospirillaceae* |
| 359953 | 2.25 | 1.32 | 0.64 | 1.18 | 0.80 | 3.66 | *Rhodobacteraceae* |
| 823476 | 2.10 | 1.20 | 0.60 | 1.21 | 0.76 | 4.42 | *Alteromonadaceae* |
| 1011088 | 1.92 | 1.72 | 0.51 | 0.98 | 0.64 | 5.06 | *Rhodobacteraceae* |
| 278985 | 0.89 | 1.66 | 0.48 | 1.25 | 0.61 | 5.67 | *Piscirickettsiaceae* |
| 589014 | 1.60 | 1.60 | 0.48 | 1.04 | 0.60 | 6.27 | *Rhodobacteraceae* |
| 548736 | 1.81 | 1.42 | 0.46 | 0.99 | 0.58 | 6.85 | *Rhodobacteraceae* |
| 1132384 | 1.55 | 1.09 | 0.46 | 1.04 | 0.58 | 7.43 | *Oceanospirillaceae* |
| 837366 | 1.00 | 1.36 | 0.46 | 0.99 | 0.58 | 8.01 | *Vibrionaceae* |
| 4214540 | 1.32 | 1.00 | 0.45 | 1.06 | 0.56 | 8.57 | *Alteromonadaceae* |
| 653071 | 0.00 | 1.21 | 0.45 | 1.40 | 0.56 | 9.13 | *Methylophilaceae* |
| 368902 | 1.21 | 1.22 | 0.44 | 1.11 | 0.56 | 9.69 | *Rhodobacteraceae* |
| 609089 | 1.14 | 1.61 | 0.44 | 0.99 | 0.55 | 10.24 | *Rhodobacteraceae* |
| 353552 | 1.16 | 0.69 | 0.44 | 0.79 | 0.55 | 10.79 | Methylophilaceae |
| 755271 | 1.13 | 0.64 | 0.42 | 1.06 | 0.53 | 11.32 | Flavobacteriaceae |
| 70494 | 1.36 | 0.80 | 0.42 | 1.21 | 0.52 | 11.84 | Unclassified, Oceanospirillales |
| 808031 | 1.18 | 0.55 | 0.42 | 1.08 | 0.52 | 12.37 | Pseudoalteromonadaceae |
| 306945 | 1.16 | 0.43 | 0.42 | 1.10 | 0.52 | 12.89 | Rhodobacteraceae |
| 549506 | 1.24 | 1.45 | 0.41 | 1.02 | 0.52 | 13.40 | Rhodobacteraceae |
| 700215 | 1.11 | 1.24 | 0.40 | 1.04 | 0.51 | 13.91 | Rhodobacteraceae |
| 330888 | 1.18 | 1.20 | 0.40 | 1.00 | 0.50 | 14.41 | Oleiphilaceae |
| 1110665 | 0.46 | 1.15 | 0.40 | 1.12 | 0.50 | 14.91 | Hyphomonadaceae |
| 887059 | 0.64 | 0.38 | 0.39 | 0.70 | 0.49 | 15.41 | Moraxellaceae |
| 512469 | 0.41 | 1.16 | 0.39 | 1.21 | 0.49 | 15.90 | Hyphomonadaceae |
| 621743 | 1.05 | 0.77 | 0.38 | 1.15 | 0.48 | 16.38 | Cryomorphaceae |
| 4390091 | 1.02 | 0.23 | 0.38 | 0.99 | 0.48 | 16.86 | Unclassified, Alteromonadales |
| 546187 | 1.15 | 1.27 | 0.38 | 1.01 | 0.47 | 17.33 | Rhodobacteraceae |
| 570128 | 0.20 | 1.13 | 0.38 | 1.33 | 0.47 | 17.80 | Piscirickettsiaceae |
| New.CleanUp.ReferenceOTU33511 | 1.00 | 0.00 | 0.37 | 0.95 | 0.47 | 18.27 | Bacteriovoracaceae |
| 537954 | 1.04 | 0.85 | 0.37 | 1.07 | 0.46 | 18.73 | Rhodobacteraceae |
| New.ReferenceOTU43 | 0.00 | 1.06 | 0.36 | 0.91 | 0.46 | 19.19 | Desulfovibrionaceae |
| New.CleanUp.ReferenceOTU18005 | 0.17 | 1.05 | 0.36 | 1.25 | 0.45 | 19.63 | Unclassified, Spirobacillales |
| New.ReferenceOTU138 | 0.57 | 0.67 | 0.35 | 0.80 | 0.44 | 20.08 | Unclassified, Flavobacteriales |

*Average dissimilarity between L4 and spat: 78.76%*

| OTU | Av. Abund (L4) | Av. Abund (Spat) | Av. Diss | Diss /SD | % Contrib | Cum. % | Taxonomy |
| --- | --- | --- | --- | --- | --- | --- | --- |
| 823160 | 1.09 | 2.66 | 0.84 | 1.30 | 1.07 | 1.07 | Piscirickettsiaceae |
| 1091363 | 0.91 | 2.06 | 0.63 | 1.33 | 0.80 | 1.87 | Methylophilaceae |
| 837366 | 1.20 | 1.36 | 0.59 | 0.74 | 0.75 | 2.62 | Vibrionaceae |
| 589014 | 1.65 | 1.60 | 0.57 | 1.08 | 0.72 | 3.34 | Rhodobacteraceae |
| 359953 | 2.08 | 1.32 | 0.56 | 1.15 | 0.71 | 4.04 | Rhodobacteraceae |
| 278985 | 1.42 | 1.66 | 0.49 | 1.04 | 0.63 | 4.67 | Piscirickettsiaceae |
| 546187 | 1.58 | 1.27 | 0.48 | 1.07 | 0.61 | 5.28 | Rhodobacteraceae |
| 548736 | 1.57 | 1.42 | 0.47 | 0.97 | 0.60 | 5.88 | Rhodobacteraceae |
| 1011088 | 1.82 | 1.72 | 0.47 | 0.98 | 0.60 | 6.49 | Rhodobacteraceae |
| 887059 | 0.79 | 0.38 | 0.47 | 0.71 | 0.59 | 7.08 | Moraxellaceae |
| 1132384 | 1.31 | 1.09 | 0.46 | 0.98 | 0.59 | 7.67 | Oceanospirillaceae |
| 823476 | 1.29 | 1.20 | 0.46 | 1.14 | 0.59 | 8.25 | Alteromonadaceae |
| 808031 | 1.10 | 0.55 | 0.45 | 0.90 | 0.57 | 8.82 | Pseudoalteromonadaceae |
| 755271 | 1.17 | 0.64 | 0.44 | 1.07 | 0.56 | 9.38 | Flavobacteriaceae |
| 609089 | 1.34 | 1.61 | 0.44 | 0.96 | 0.56 | 9.95 | Rhodobacteraceae |
| 653071 | 0.34 | 1.21 | 0.44 | 1.25 | 0.55 | 10.50 | Methylophilaceae |
| 368902 | 1.37 | 1.22 | 0.43 | 1.06 | 0.55 | 11.05 | Rhodobacteraceae |
| 549506 | 1.52 | 1.45 | 0.43 | 0.97 | 0.55 | 11.60 | Rhodobacteraceae |
| 330888 | 1.01 | 1.20 | 0.43 | 1.03 | 0.54 | 12.15 | Oleiphilaceae |
| 248432 | 1.09 | 0.93 | 0.42 | 1.11 | 0.54 | 12.68 | Oceanospirillaceae |
| 1078207 | 0.60 | 0.38 | 0.41 | 0.63 | 0.52 | 13.20 | Streptococcaceae |
| 700215 | 0.95 | 1.24 | 0.40 | 1.06 | 0.51 | 13.72 | Rhodobacteraceae |
| 512469 | 0.58 | 1.16 | 0.40 | 1.19 | 0.51 | 14.23 | Hyphomonadaceae |
| 1110665 | 0.59 | 1.15 | 0.40 | 1.07 | 0.51 | 14.74 | Hyphomonadaceae |
| 572325 | 0.72 | 1.27 | 0.40 | 1.19 | 0.50 | 15.24 | Rhodobacteraceae |
| 941487 | 0.48 | 0.41 | 0.38 | 0.66 | 0.49 | 15.73 | Oxalobacteraceae |
| 570128 | 0.53 | 1.13 | 0.38 | 1.22 | 0.48 | 16.21 | Piscirickettsiaceae |
| 4214540 | 0.70 | 1.00 | 0.37 | 1.16 | 0.47 | 16.68 | Alteromonadaceae |
| New.ReferenceOTU43 | 0.00 | 1.06 | 0.37 | 0.91 | 0.47 | 17.15 | Desulfovibrionaceae |
| 1101488 | 0.83 | 0.98 | 0.37 | 1.08 | 0.47 | 17.62 | Rhodobacteraceae |
| 665721 | 0.56 | 1.04 | 0.37 | 1.14 | 0.47 | 18.09 | Piscirickettsiaceae |
| New.CleanUp.ReferenceOTU18005 | 0.18 | 1.05 | 0.36 | 1.25 | 0.46 | 18.55 | Unclassified, Spirobacillales |
| 1105814 | 0.51 | 0.27 | 0.36 | 0.54 | 0.46 | 19.01 | Bradyrhizobiaceae |
| 2932342 | 0.00 | 1.00 | 0.36 | 0.79 | 0.45 | 19.46 | Desulfovibrionaceae |
| 899488 | 0.80 | 0.80 | 0.36 | 1.05 | 0.45 | 19.91 | Alteromonadaceae |
| New.ReferenceOTU138 | 0.52 | 0.67 | 0.35 | 0.78 | 0.45 | 20.36 | Unclassified, Flavobacteriales |

*Average dissimilarity between L2 and juvenile: 92.68%*

| OTU | Av.  Abund (L2) | Av. Abund (Juv) | Av. Diss | Diss /SD | % Contrib | Cum. % | Taxonomy |
| --- | --- | --- | --- | --- | --- | --- | --- |
| 217851 | 0.05 | 3.53 | 1.17 | 2.60 | 1.26 | 1.26 | Simkaniaceae |
| 824245 | 0.00 | 2.14 | 0.71 | 3.73 | 0.77 | 2.03 | Unclassified, Rhizobiales |
| 248432 | 2.13 | 0.00 | 0.67 | 1.90 | 0.73 | 2.76 | Oceanospirillaceae |
| New.ReferenceOTU110 | 0.00 | 1.97 | 0.65 | 4.31 | 0.71 | 3.46 | Unclassified, Rhodospirillales |
| New.ReferenceOTU161 | 0.00 | 1.93 | 0.65 | 2.40 | 0.70 | 4.17 | Gloeobacteraceae |
| 823476 | 2.10 | 0.17 | 0.63 | 1.77 | 0.68 | 4.84 | Alteromonadaceae |
| 359953 | 2.25 | 0.65 | 0.61 | 1.80 | 0.66 | 5.50 | Rhodobacteraceae |
| 331011 | 0.00 | 1.80 | 0.61 | 2.91 | 0.65 | 6.15 | Phyllobacteriaceae |
| 428807 | 0.00 | 1.80 | 0.59 | 4.60 | 0.64 | 6.79 | Hyphomicrobiaceae |
| New.ReferenceOTU118 | 0.00 | 1.66 | 0.56 | 2.39 | 0.60 | 7.39 | Gloeobacteraceae |
| 1119126 | 0.00 | 1.59 | 0.53 | 3.07 | 0.57 | 7.96 | Unclassified, Alphaproteobacteria |
| 587505 | 0.00 | 1.57 | 0.53 | 2.47 | 0.57 | 8.53 | Rhodospirillaceae |
| 325677 | 0.00 | 1.50 | 0.50 | 2.75 | 0.54 | 9.07 | Unclassified, Rhizobiales |
| 579649 | 0.00 | 1.49 | 0.49 | 3.93 | 0.53 | 9.60 | Unclassified, Rhodospirillales |
| 194304 | 0.00 | 1.43 | 0.48 | 1.77 | 0.52 | 10.12 | Xenococcaceae |
| New.ReferenceOTU79 | 0.00 | 1.44 | 0.48 | 2.31 | 0.52 | 10.64 | Amoebophilaceae |
| 1132384 | 1.55 | 0.00 | 0.47 | 1.52 | 0.51 | 11.15 | Oceanospirillaceae |
| 1011088 | 1.92 | 0.96 | 0.47 | 2.68 | 0.51 | 11.66 | Rhodobacteraceae |
| 548736 | 1.81 | 0.60 | 0.45 | 1.70 | 0.49 | 12.15 | Rhodobacteraceae |
| New.ReferenceOTU155 | 0.00 | 1.35 | 0.44 | 1.38 | 0.47 | 12.62 | Nostocaceae |
| 818655 | 0.00 | 1.33 | 0.44 | 4.49 | 0.47 | 13.10 | Pirellulaceae |
| New.ReferenceOTU148 | 0.00 | 1.33 | 0.43 | 1.25 | 0.47 | 13.56 | Unclassified, Nostocophycideae |
| 4396978 | 0.00 | 1.30 | 0.43 | 2.63 | 0.46 | 14.03 | Phyllobacteriaceae |
| 4381005 | 0.00 | 1.26 | 0.42 | 2.26 | 0.45 | 14.48 | Rhodobacteraceae |
| 70494 | 1.36 | 0.00 | 0.41 | 1.54 | 0.44 | 14.92 | Unclassified, Oceanospirillales |
| 4214540 | 1.32 | 0.05 | 0.40 | 1.13 | 0.44 | 15.36 | Alteromonadaceae |
| 1119959 | 0.00 | 1.21 | 0.40 | 2.43 | 0.44 | 15.79 | Hyphomicrobiaceae |
| 792011 | 0.00 | 1.21 | 0.40 | 2.61 | 0.43 | 16.22 | Unclassified, Myxococcales |
| 350435 | 0.00 | 1.22 | 0.40 | 2.33 | 0.43 | 16.66 | JdFBGBact |
| 2357381 | 0.00 | 1.21 | 0.40 | 1.87 | 0.43 | 17.09 | Unclassified, Rhizobiales |
| 1753261 | 0.00 | 1.23 | 0.40 | 1.86 | 0.43 | 17.52 | Flavobacteriaceae |
| New.CleanUp.ReferenceOTU9481 | 0.00 | 1.12 | 0.38 | 1.90 | 0.41 | 17.93 | Simkaniaceae |
| 353552 | 1.16 | 0.00 | 0.37 | 0.97 | 0.40 | 18.33 | Methylophilaceae |
| 831558 | 0.00 | 1.14 | 0.37 | 1.40 | 0.40 | 18.74 | Endozoicimonaceae |
| New.CleanUp.ReferenceOTU27229 | 0.00 | 1.13 | 0.37 | 1.93 | 0.40 | 19.14 | Unclassified, Rhodospirillales |
| 808031 | 1.18 | 0.00 | 0.36 | 1.19 | 0.39 | 19.53 | Pseudoalteromonadaceae |
| 45178 | 0.00 | 1.06 | 0.36 | 1.94 | 0.39 | 19.92 | Xenococcaceae |
| 583197 | 0.00 | 1.08 | 0.36 | 1.23 | 0.38 | 20.31 | Amoebophilaceae |

*Average dissimilarity between L4 and juvenile: 92.20%*

| OTU | Av. Abund (L4) | Av. Abund (Juv) | Av. Diss | Diss /SD | % Contrib | Cum. % | Taxonomy |
| --- | --- | --- | --- | --- | --- | --- | --- |
| 217851 | 0.07 | 3.53 | 1.19 | 2.52 | 1.29 | 1.29 | Simkaniaceae |
| 824245 | 0.00 | 2.14 | 0.73 | 3.70 | 0.79 | 2.08 | Unclassified, Rhizobiales |
| New.ReferenceOTU161 | 0.06 | 1.93 | 0.65 | 2.23 | 0.71 | 2.79 | Gloeobacteraceae |
| New.ReferenceOTU110 | 0.12 | 1.97 | 0.63 | 3.29 | 0.68 | 3.47 | Unclassified, Rhodospirillales |
| 331011 | 0.00 | 1.80 | 0.62 | 2.88 | 0.67 | 4.14 | Phyllobacteriaceae |
| 428807 | 0.00 | 1.80 | 0.61 | 4.55 | 0.66 | 4.80 | Hyphomicrobiaceae |
| New.ReferenceOTU118 | 0.00 | 1.66 | 0.57 | 2.38 | 0.62 | 5.42 | Gloeobacteraceae |
| 359953 | 2.08 | 0.65 | 0.54 | 1.77 | 0.59 | 6.00 | Rhodobacteraceae |
| 587505 | 0.00 | 1.57 | 0.54 | 2.46 | 0.58 | 6.58 | Rhodospirillaceae |
| 1119126 | 0.07 | 1.59 | 0.52 | 2.69 | 0.56 | 7.15 | Unclassified, Alphaproteobacteria |
| 325677 | 0.00 | 1.50 | 0.51 | 2.75 | 0.55 | 7.70 | Unclassified, Rhizobiales |
| 579649 | 0.00 | 1.49 | 0.50 | 3.90 | 0.54 | 8.24 | Unclassified, Rhodospirillales |
| New.ReferenceOTU79 | 0.00 | 1.44 | 0.49 | 2.30 | 0.53 | 8.78 | Amoebophilaceae |
| 546187 | 1.58 | 0.06 | 0.49 | 1.52 | 0.53 | 9.31 | Rhodobacteraceae |
| 194304 | 0.12 | 1.43 | 0.46 | 1.60 | 0.50 | 9.81 | Xenococcaceae |
| 818655 | 0.00 | 1.33 | 0.45 | 4.45 | 0.49 | 10.30 | Pirellulaceae |
| New.ReferenceOTU148 | 0.00 | 1.33 | 0.44 | 1.25 | 0.48 | 10.78 | Unclassified, Nostocophycideae |
| New.ReferenceOTU155 | 0.06 | 1.35 | 0.44 | 1.39 | 0.48 | 11.26 | Nostocaceae |
| 4396978 | 0.00 | 1.30 | 0.44 | 2.62 | 0.48 | 11.73 | Phyllobacteriaceae |
| 278985 | 1.42 | 0.00 | 0.44 | 1.32 | 0.48 | 12.21 | Piscirickettsiaceae |
| 549506 | 1.52 | 0.31 | 0.44 | 1.54 | 0.47 | 12.68 | Rhodobacteraceae |
| 4381005 | 0.00 | 1.26 | 0.43 | 2.25 | 0.47 | 13.14 | Rhodobacteraceae |
| 548736 | 1.57 | 0.60 | 0.42 | 1.63 | 0.46 | 13.60 | Rhodobacteraceae |
| 1119959 | 0.00 | 1.21 | 0.41 | 2.42 | 0.45 | 14.05 | Hyphomicrobiaceae |
| 1132384 | 1.31 | 0.00 | 0.41 | 1.19 | 0.45 | 14.50 | Oceanospirillaceae |
| 589014 | 1.65 | 1.88 | 0.41 | 1.38 | 0.45 | 14.94 | Rhodobacteraceae |
| 792011 | 0.00 | 1.21 | 0.41 | 2.60 | 0.44 | 15.39 | Unclassified, Myxococcales |
| 350435 | 0.00 | 1.22 | 0.41 | 2.33 | 0.44 | 15.83 | JdFBGBact |
| 837366 | 1.20 | 0.67 | 0.41 | 0.75 | 0.44 | 16.28 | Vibrionaceae |
| 2357381 | 0.00 | 1.21 | 0.41 | 1.86 | 0.44 | 16.72 | Unclassified, Rhizobiales |
| 1753261 | 0.00 | 1.23 | 0.41 | 1.85 | 0.44 | 17.16 | Flavobacteriaceae |
| New.CleanUp.ReferenceOTU9481 | 0.00 | 1.12 | 0.39 | 1.90 | 0.42 | 17.58 | Simkaniaceae |
| 823476 | 1.29 | 0.17 | 0.38 | 1.22 | 0.42 | 18.00 | Alteromonadaceae |
| 1011088 | 1.82 | 0.96 | 0.38 | 1.96 | 0.42 | 18.42 | Rhodobacteraceae |
| 831558 | 0.00 | 1.14 | 0.38 | 1.40 | 0.42 | 18.83 | Endozoicimonaceae |
| New.CleanUp.ReferenceOTU27229 | 0.00 | 1.13 | 0.38 | 1.93 | 0.41 | 19.25 | Unclassified, Rhodospirillales |
| 368902 | 1.37 | 0.55 | 0.37 | 1.43 | 0.40 | 19.65 | Rhodobacteraceae |
| 583197 | 0.00 | 1.08 | 0.36 | 1.23 | 0.40 | 20.04 | Amoebophilaceae |

*Average dissimilarity between spat and juvenile: 86.98%*

| OTU | Av. Abund (Spat) | Av. Abund (Juv) | Av. Diss | Diss/SD | % Contrib | Cum. % | Taxonomy |
| --- | --- | --- | --- | --- | --- | --- | --- |
| 217851 | 0.07 | 3.53 | 1.02 | 2.51 | 1.17 | 1.17 | Simkaniaceae |
| 823160 | 2.66 | 0.00 | 0.74 | 1.79 | 0.85 | 2.02 | Piscirickettsiaceae |
| New.ReferenceOTU161 | 0.11 | 1.93 | 0.54 | 2.04 | 0.62 | 2.64 | Gloeobacteraceae |
| 1091363 | 2.06 | 0.05 | 0.54 | 1.76 | 0.62 | 3.26 | Methylophilaceae |
| 824245 | 0.38 | 2.14 | 0.53 | 2.01 | 0.61 | 3.87 | Unclassified, Rhizobiales |
| New.ReferenceOTU110 | 0.23 | 1.97 | 0.51 | 2.67 | 0.59 | 4.45 | Unclassified, Rhodospirillales |
| New.ReferenceOTU118 | 0.00 | 1.66 | 0.49 | 2.30 | 0.56 | 5.02 | Unclassified, Rhodospirillales |
| 587505 | 0.00 | 1.57 | 0.46 | 2.36 | 0.53 | 5.55 | Rhodospirillaceae |
| 331011 | 0.29 | 1.80 | 0.46 | 1.88 | 0.53 | 6.07 | Phyllobacteriaceae |
| 428807 | 0.31 | 1.80 | 0.45 | 2.11 | 0.51 | 6.59 | Hyphomicrobiaceae |
| 278985 | 1.66 | 0.00 | 0.44 | 1.69 | 0.51 | 7.09 | Piscirickettsiaceae |
| 194304 | 0.00 | 1.43 | 0.42 | 1.75 | 0.49 | 7.58 | Xenococcaceae |
| 579649 | 0.11 | 1.49 | 0.40 | 2.57 | 0.46 | 8.04 | Unclassified, Rhodospirillales |
| 325677 | 0.21 | 1.50 | 0.40 | 1.93 | 0.45 | 8.50 | Unclassified, Rhizobiales |
| New.ReferenceOTU79 | 0.18 | 1.44 | 0.39 | 1.83 | 0.45 | 8.94 | Amoebophilaceae |
| New.ReferenceOTU148 | 0.00 | 1.33 | 0.38 | 1.23 | 0.44 | 9.38 | Unclassified, Nostocophycideae |
| 4396978 | 0.00 | 1.30 | 0.38 | 2.50 | 0.43 | 9.81 | Phyllobacteriaceae |
| New.ReferenceOTU155 | 0.06 | 1.35 | 0.38 | 1.39 | 0.43 | 10.25 | Nostocaceae |
| 217851 | 0.07 | 3.53 | 1.02 | 2.51 | 1.17 | 1.17 | Simkaniaceae |
| 818655 | 0.06 | 1.33 | 0.37 | 3.08 | 0.43 | 10.68 | Pirellulaceae |
| 549506 | 1.45 | 0.31 | 0.35 | 1.55 | 0.40 | 11.07 | Rhodobacteraceae |
| 792011 | 0.06 | 1.21 | 0.34 | 2.20 | 0.39 | 11.46 | Unclassified, Myxococcales |
| 1011088 | 1.72 | 0.96 | 0.34 | 2.32 | 0.39 | 11.85 | Rhodobacteraceae |
| 1119959 | 0.06 | 1.21 | 0.34 | 2.23 | 0.39 | 12.24 | Hyphomicrobiaceae |
| New.CleanUp.ReferenceOTU9481 | 0.00 | 1.12 | 0.33 | 1.85 | 0.38 | 12.62 | Simkaniaceae |
| 831558 | 0.00 | 1.14 | 0.33 | 1.37 | 0.38 | 13.00 | Endozoicimonaceae |
| 653071 | 1.21 | 0.00 | 0.33 | 1.57 | 0.37 | 13.37 | Methylophilaceae |
| 330888 | 1.20 | 0.11 | 0.32 | 1.39 | 0.37 | 13.75 | Oleiphilaceae |
| 546187 | 1.27 | 0.06 | 0.32 | 1.47 | 0.37 | 14.12 | Rhodobacteraceae |
| New.CleanUp.ReferenceOTU27229 | 0.07 | 1.13 | 0.32 | 1.75 | 0.37 | 14.48 | Unclassified, Rhodospirillales |
| 350435 | 0.19 | 1.22 | 0.32 | 1.73 | 0.37 | 14.85 | JdFBGBact |
| 548736 | 1.42 | 0.60 | 0.32 | 1.35 | 0.36 | 15.21 | Rhodobacteraceae |
| 583197 | 0.09 | 1.08 | 0.31 | 1.20 | 0.36 | 15.57 | Amoebophilaceae |
| New.CleanUp.ReferenceOTU12934 | 0.00 | 1.05 | 0.31 | 1.61 | 0.36 | 15.93 | Simkaniaceae |
| 1753261 | 0.32 | 1.23 | 0.31 | 1.45 | 0.35 | 16.28 | Flavobacteriaceae |
| 1119126 | 0.73 | 1.59 | 0.31 | 1.30 | 0.35 | 16.63 | Unclassified, Alphaproteobacteria |
| 589014 | 1.60 | 1.88 | 0.30 | 1.07 | 0.35 | 16.98 | Rhodobacteraceae |
| 2357381 | 0.35 | 1.21 | 0.30 | 1.40 | 0.35 | 17.33 | Unclassified, Rhizobiales |
| 823476 | 1.20 | 0.17 | 0.30 | 1.44 | 0.35 | 17.68 | Alteromonadaceae |
| 321503 | 0.06 | 1.04 | 0.30 | 1.44 | 0.34 | 18.02 | Pirellulaceae |
| 4381005 | 0.39 | 1.26 | 0.30 | 1.42 | 0.34 | 18.36 | Rhodobacteraceae |
| 4371752 | 0.07 | 1.02 | 0.30 | 1.37 | 0.34 | 18.70 | Unclassified, Kiloniellales |
| 342034 | 0.00 | 1.03 | 0.30 | 1.40 | 0.34 | 19.04 | Unclassified, Alphaproteobacteria |
| New.CleanUp.ReferenceOTU8909 | 0.07 | 1.04 | 0.30 | 1.53 | 0.34 | 19.38 | Unclassified, Rhizobiales |
| 842393 | 0.00 | 0.98 | 0.29 | 1.38 | 0.34 | 19.72 | Xenococcaceae |
| 570128 | 1.13 | 0.00 | 0.29 | 1.48 | 0.34 | 20.05 | Piscirickettsiaceae |
